# Advances in the biomimetic and Maillard route synthesis and regulation of pyrazine flavour compounds

**DOI:** 10.1039/d6ra07015a

**Published:** 2026-10-22

**Authors:** Bingyang Wang, Yao Zhao, Lianchen Zhao, Wentao Zhu, Jie Ren

**Affiliations:** a School of Tobacco Science and Engineering, Zhengzhou University of Light Industry No. 136 Ke Xue Avenue Zhengzhou Henan 450002 People's Republic of China 2021038@zzuli.edu.cn

## Abstract

Pyrazines are nitrogen-containing heterocyclic flavour compounds characterized by roasted, nutty, and coffee-like aroma notes, and play a key role in the flavour quality of thermally processed foods. Biomimetic and Maillard route synthesis enables efficient generation of pyrazine flavourants from readily available、renewable natural precursors, offering green and sustainable advantages. This review systematically summarizes the research progress in this field since the 1960s, following three main threads: precursor types, formation mechanisms, and regulatory strategies. It reviews the pyrazine-forming capabilities of various precursors including small-molecule carbonyl compounds, sugar-amino acid/ammonia systems, hydroxy amino acids, lipid degradation products, ascorbic acid, and peptides/proteins. Multiple coexisting mechanistic pathways are elucidated, including α-aminoketone condensation-oxidation, free radical pathways, azomethine ylide dimerisation, and peptide-involved mechanisms. The regulatory effects of temperature, pH, precursor structure, reaction media, and emerging technologies on pyrazine yield and distribution are summarized, and the application of computational chemistry in mechanistic studies is discussed. Finally, future research directions are prospected from the perspectives of peptide structure–product relationships, multi-component network analysis, data-driven synthesis, and engineering scale-up, aiming to provide theoretical guidance for the design and efficient preparation of pyrazine flavourants.

## Abstract 2

This review summarizes the biomimetic and Maillard route synthesis of pyrazine flavour compounds, covering precursor types, formation mechanisms, and regulatory strategies, and highlighting future directions towards sustainable manufacturing.

## Graphical abstract

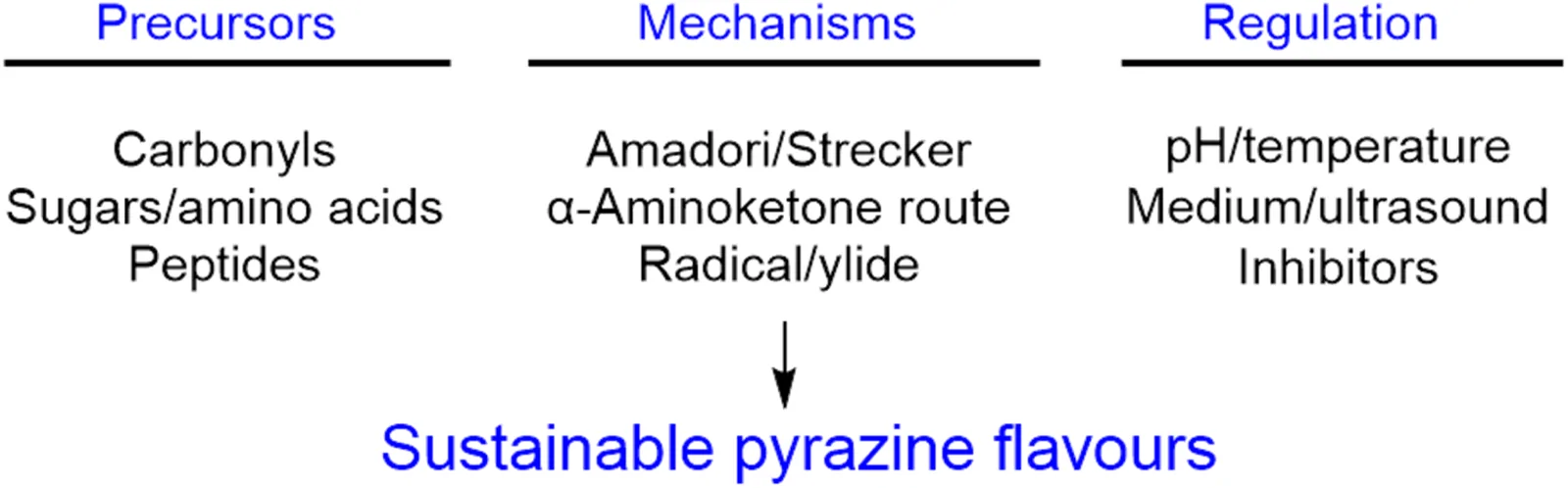

## 1. Introduction

Pyrazines are a class of heterocyclic compounds with roasted, nutty, and coffee-like aroma notes, widely present in thermally processed foods and serving as important contributors to characteristic food flavours. In 1966, Dawes and Edwards first confirmed that pyrazines could be generated *via* the Maillard reaction by heating sugar–amino acid mixtures.^1^ Subsequently, Mason *et al.*, Bondarovich *et al.*, and Wang *et al.* identified pyrazines in roasted peanuts, coffee, and barley, respectively, and verified through glyoxal–amino acid model reactions the pathway of α-dicarbonyl compound condensation with amino acids to form pyrazines.^2–4^ These early studies established pyrazines as key flavour compounds in thermally processed foods. Maga systematically reviewed the distribution and aroma characteristics of pyrazines in various foods, noting that the odor thresholds of alkyl-substituted pyrazines can be as low as the µg L^−1^ or even ng L^−1^ level.^5,6^ Building on this foundation, researchers began to systematically explore the formation mechanisms and synthetic pathways of pyrazines, leading to the development of a series of biomimetic synthesis strategies.

The Maillard reaction is a complex reaction network between reducing sugars and amino compounds, with pyrazine formation primarily dependent on the condensation, cyclisation, and oxidation of α-dicarbonyl compounds derived from sugar degradation with amino acid-derived nitrogen. Biomimetic and Maillard route synthesis utilizes inexpensive and renewable natural precursors to efficiently generate pyrazine flavour compounds under simulated natural processing conditions, offering green and sustainable advantages. In recent years, Yu *et al.* and Zhou *et al.* have reviewed the research progress on pyrazine generation from the perspectives of broad regulatory strategies and peptide specificity, respectively; Ren *et al.* have provided technical support for the precise identification of pyrazines from the perspective of analytical methods.^7–9^ However, existing reviews have not integrated precursor types, formation mechanisms, and regulatory strategies as a coherent logical thread. In this review, “biomimetic synthesis” refers specifically to the chemical simulation of Maillard reaction networks during food thermal processing, rather than enzymatic or microbial pathways. Within this defined scope, we take biomimetic synthesis as our core perspective and systematically summarize the progress in pyrazine flavour compound synthesis through three progressive levels—precursor sources → formation mechanisms → regulatory strategies—aiming to provide theoretical guidance for the design and efficient preparation of pyrazine flavourants.

## 2. Precursors for pyrazine formation

The Maillard reaction formation of pyrazines depends on the presence of specific chemical precursors. The type and structure of precursors not only determine the efficiency of pyrazine generation but also directly influence the substitution patterns and aroma characteristics of the products.

### 2.1 Direct condensation of small-molecule carbonyl compounds with ammonia/amino acids

Koehler and Odell systematically investigated the reactions of α-dicarbonyl compounds (glyoxal, methylglyoxal, butanedione) and α-hydroxycarbonyl compounds (hydroxyacetone) with ammonia or amino acids, and found that these precursors could directly condense to form pyrazines with high yields (Table 1).^10^

**Table 1:** Yields and selectivity of pyrazine formation from small-molecule carbonyl compounds with ammonia or amino acids

Precursor	Reactant	Major product	Yield (µmol/220 mL)	Selectivity
2,3-Butanedione	Asparagine	Tetramethylpyrazine	2662	Formed exclusively; no other alkylpyrazines detected
Hydroxyacetone	Ammonia or asparagine	Dimethylpyrazine	9725	Extremely high yield; almost exclusively dimethylpyrazine
Glyoxal	Asparagine	Pyrazine	527	Minor methylpyrazine formed as by-product
Acetaldehyde	Ammonia	Pyrazine	399	No alkylpyrazines formed

The reactivity of different precursors varied significantly: 2,3-butanedione, as a complete C_4_ fragment, could directly participate in pyrazine ring construction without cleavage, yielding tetramethylpyrazine with high specificity. Hydroxyacetone exhibited a far higher yield than other precursors, serving as a key intermediate for dimethylpyrazine formation. Acetaldehyde reacted with asparagine to generate methyl-, dimethyl-, and trimethylpyrazines, whereas its reaction with ammonia yielded only pyrazine, indicating that amino acids promoted carbon chain elongation. Propanal did not produce any pyrazine under the same conditions, suggesting that not all aldehydes can effectively participate in pyrazine formation. The work of Koehler and Odell was the first to quantitatively reveal that small-molecule carbonyl compounds derived from sugar degradation could be efficiently and selectively converted into specific pyrazines.

### 2.2 Sugar–amino acid/ammonia model systems

The Maillard reaction between reducing sugars and amino compounds begins with the condensation of the carbonyl group of an aldose or ketose with the amino group of an amino acid or ammonia to form a Schiff base, which cyclises to an *N*-substituted glycosylamine and then undergoes the Amadori rearrangement to yield the key 1-amino-1-deoxy-2-ketose (Amadori rearrangement product, ARP). For ketohexoses, the analogous transformation is known as the Heyns rearrangement. These ARPs are the central intermediates that subsequently degrade into α-dicarbonyl compounds and other reactive fragments, ultimately leading to pyrazine formation (Scheme 1).

**Scheme 1:**
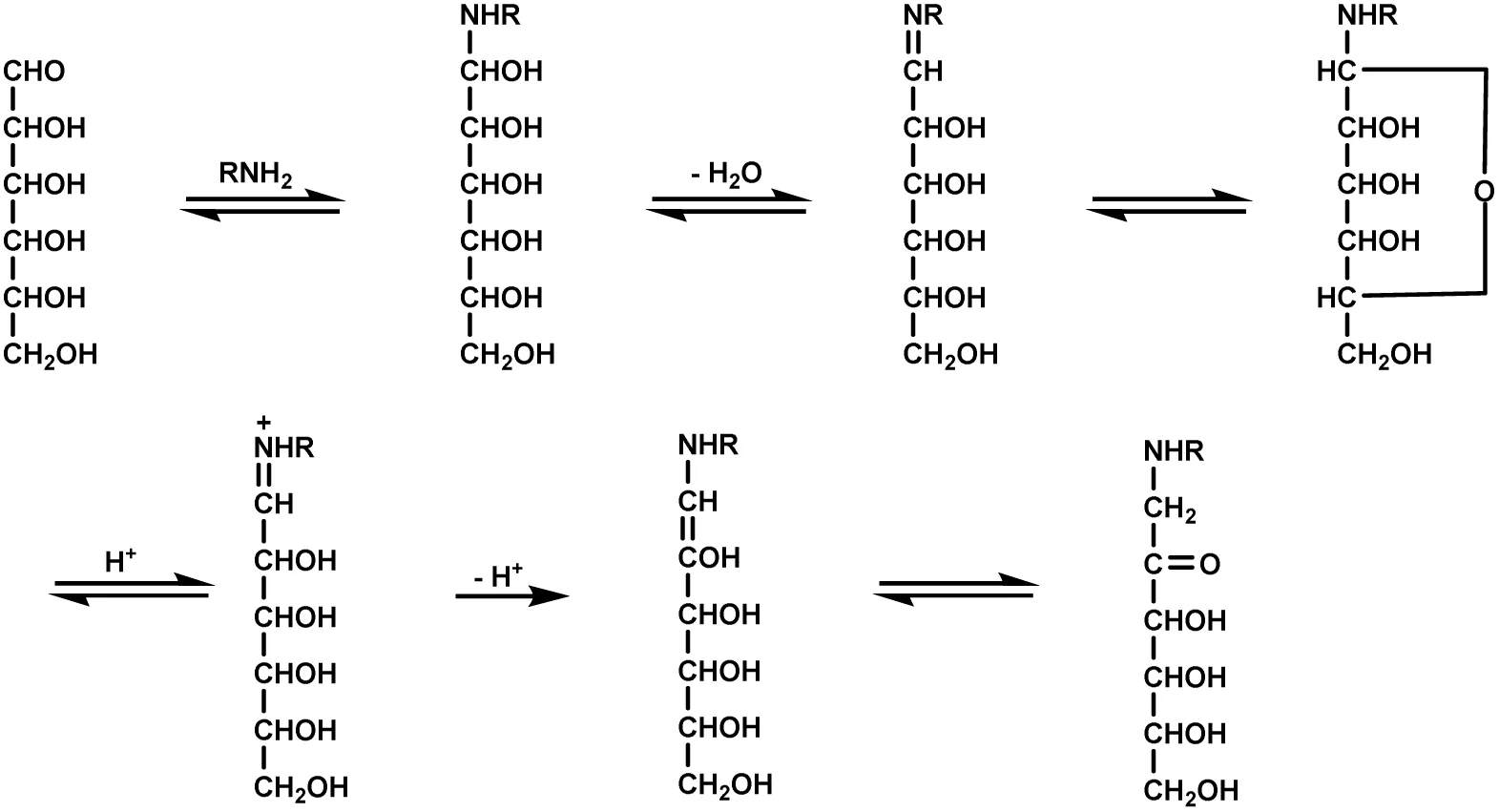
Amadori rearrangement pathway for aldoses and ketoses in the Maillard reaction. Ketohexoses undergo the analogous Heyns rearrangement (R represents an alkyl or aryl group derived from the primary amine RNH_2_).

van Praag *et al.* first compared the glucose–ammonia and rhamnose–ammonia model systems, finding that the glucose–ammonia system predominantly generated 2-methylpyrazine, while the rhamnose–ammonia system produced substantial amounts of ethyl-substituted pyrazines.^11^ Rhamnose, a 6-deoxyhexose, can directly participate in ethyl group introduction *via* its C6 methyl group without cleavage, providing the first evidence that sugar structure exerts a decisive influence on pyrazine type and distribution (Fig. 1).

**Fig. 1:**
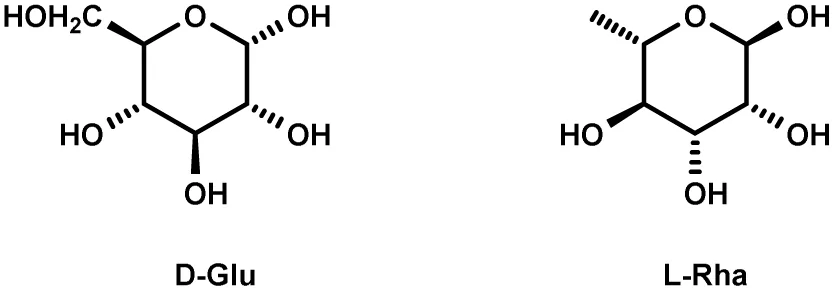
Structures of glucose and rhamnose.

Shibamoto and Bernhard examined the pyrazine distribution from the reaction of 15 carbohydrates with ammonia.^12^ Aldohexoses and ketohexoses exhibited similar distribution patterns, with 2-methylpyrazine and 2,6-dimethylpyrazine as the major products, and no significant differences were observed between stereoisomers. The total yield of 6-deoxyhexoses reached 12.5%, approximately 10 times that of common hexoses, with a marked increase in the proportion of ethyl-substituted pyrazines. 2-Deoxyglucose, lacking the C2 hydroxyl group, could not undergo Amadori rearrangement and thus showed extremely low total yield, but generated substantial amounts of 2-ethylpyrazine and 2-ethyl-5-vinylpyrazine, suggesting the existence of a retro-aldol cleavage pathway. Sugar alcohols produced almost no pyrazines, confirming the necessity of the carbonyl group; C_3_ units generated only methyl-/dimethylpyrazines, indicating that insufficient carbon chain length precludes the formation of higher alkylpyrazines. Collectively, the cleavage pattern of sugars determines the type and distribution of substituents on the pyrazine ring.

The origin of these differences lies in the distinct cleavage pathways of the sugar backbone. Sugar cleavage mainly proceeds through retro-aldol cleavage, dehydration, and enediol cleavage pathways, generating α-dicarbonyl fragments of varying carbon chain lengths. Based on these findings, Shibamoto and Bernhard proposed 10 α-aminocarbonyl intermediates and pointed out that the cleavage pattern of sugars determines the relative concentrations of these intermediates, which in turn governs the types and positions of substituents on the pyrazine ring (Table 2).^12^

**Table 2:** Correspondence between different substrate reactions and the formation of corresponding pyrazine products. (Only the pyrazine structures detected in the corresponding model systems are shown; blank cells indicate that the corresponding pyrazine was not detected or not reported.)

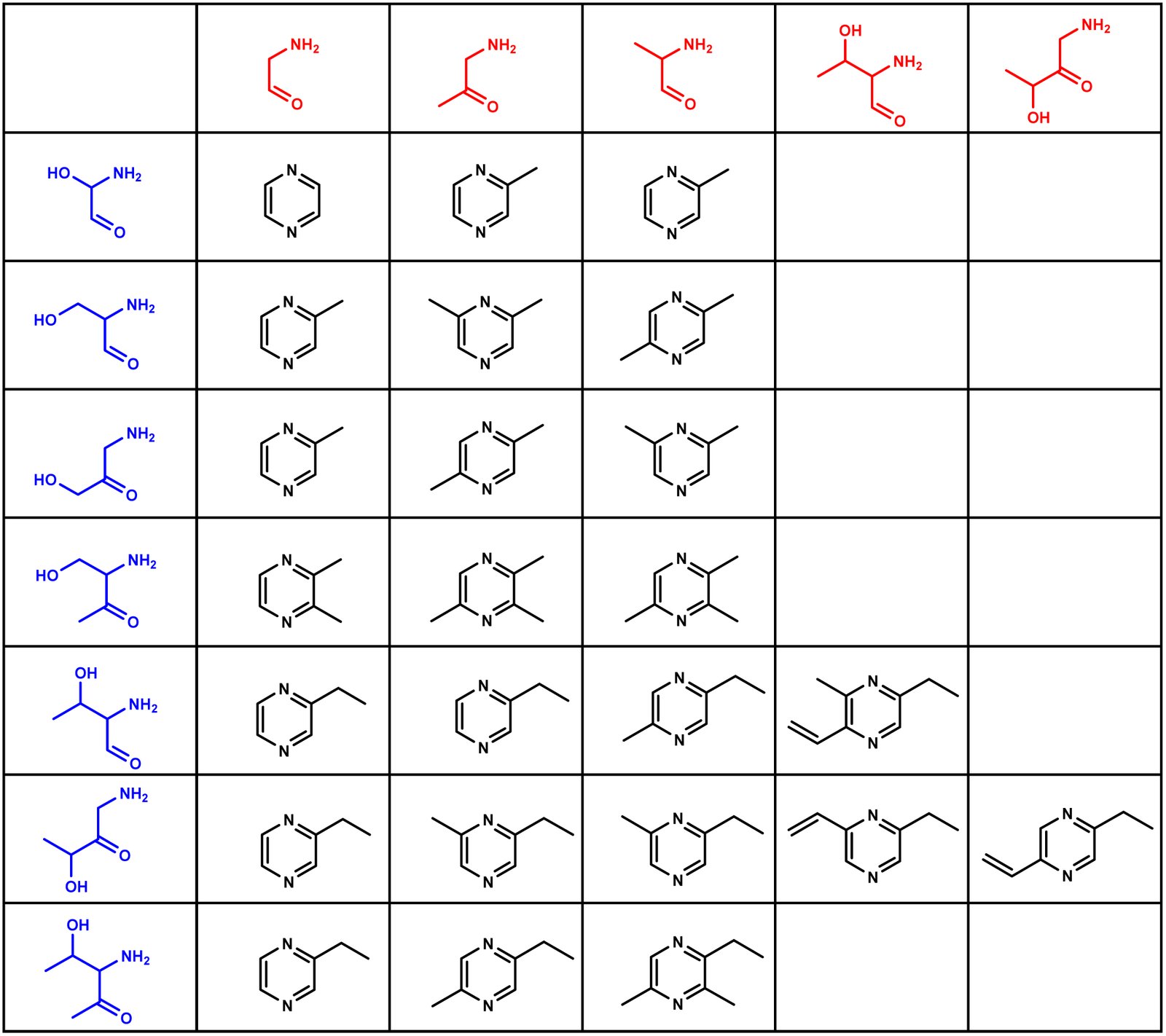

Model studies using 1,3-dihydroxyacetone (DHA) as the carbonyl source further elucidated the mechanism of substituent introduction onto the pyrazine ring. Adams *et al.* found that 2,5(6)-dimethylpyrazine was the predominant product across all reaction systems, confirming that DHA-derived 2-oxopropanal is the key precursor of the pyrazine core skeleton; the substituents of other alkylpyrazines mainly originate from the corresponding amino acid Strecker aldehydes or aldol condensation products of 2-oxopropanal.^13^ The 3,6-dimethyl-2,5-dihydropyrazine, formed by condensation of two molecules of 2-oxopropanal-derived aminoketones, can undergo nucleophilic addition with aldehydes or ketones in the system, followed by dehydration to yield 3-alkyl-substituted pyrazines. When the nucleophile is an α-dicarbonyl compound, 3-acyl-substituted pyrazines are generated. A common feature of the above pathways is that the dihydropyrazine intermediate can be directly converted to pyrazine products without undergoing an oxidation step (Scheme 2).

**Scheme 2:**
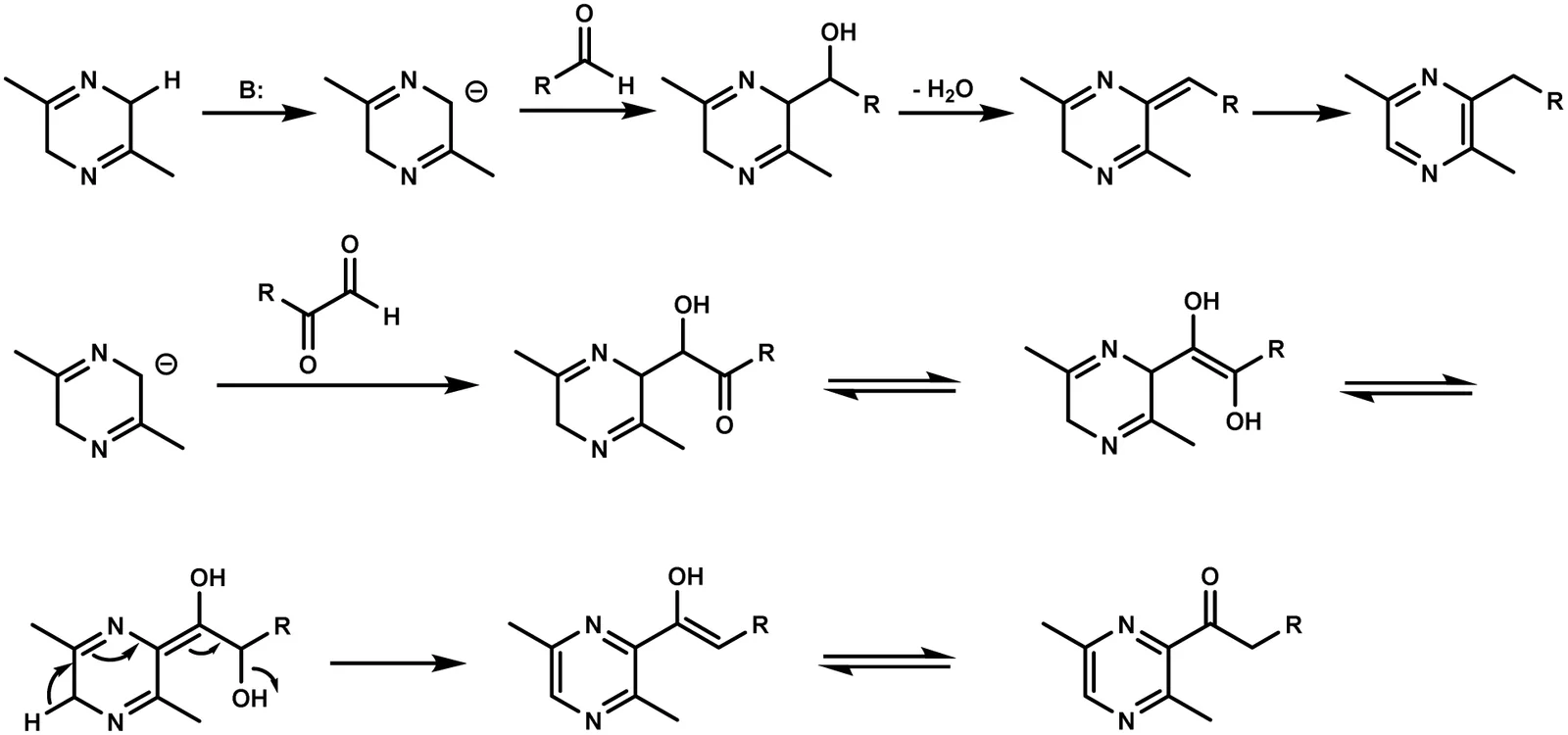
Reaction pathways of dihydropyrazine intermediates with carbonyl compounds (R represents H, alkyl, alkenyl, aryl, or heterocyclic groups).

Ferretti *et al.* identified pyrazine and various methylpyrazines in a casein–lactose dry-state Maillard reaction system, demonstrating that pyrazines remain characteristic Maillard reaction products even in complex protein systems.^14^ Hwang *et al.* employed ^15^N isotope labelling and found that the amide nitrogen of glutamine can directly participate in pyrazine ring construction through the release of free ammonia, with a contribution exceeding 50%—far higher than that of the α-amino group. This is because the amide nitrogen does not require the decarboxylation step of Strecker degradation, making the pathway more direct.^15^ This explains the early observation that the pyrazine yield from the reaction of glutamine and asparagine with sugars is significantly higher than that of their corresponding acidic amino acids.^6^ The ε-amino group of lysine can also participate in pyrazine formation, but with lower efficiency than the α-amino group: the α-amino group generates α-aminoketones *via* Strecker degradation, whereas the ε-amino group requires intramolecular rearrangement and hydration, with the additional steps reducing its contribution.^16^ Although temperature and pH significantly affect the total pyrazine yield, they do not alter the relative contribution ratio of the two amino groups (Scheme 3). These findings expand the understanding of pyrazine nitrogen sources from a single α-amino group to a multi-faceted framework involving the synergistic contribution of multiple nitrogen-containing groups.

**Scheme 3:**
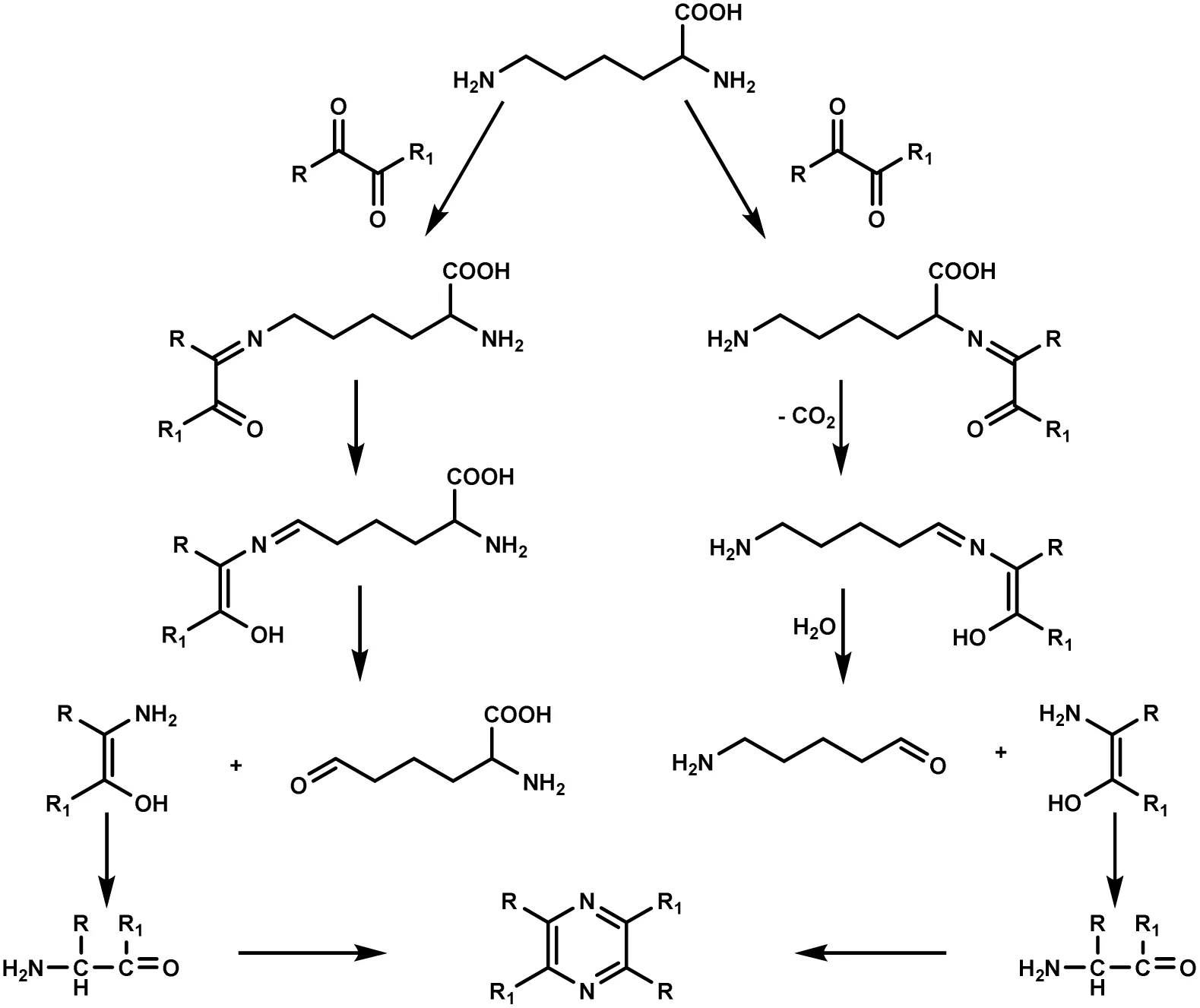
Proposed mechanisms for transamination from α-and ε-amino groups of lysine to pyrazines (R and R^1^ represent H or alkyl groups).

Although Amadori rearrangement products (ARPs) are considered key intermediates in the Maillard reaction, Deng *et al.* observed that the types and total amount of pyrazines generated from the thermal decomposition of the methionine–glucose ARP system were lower than those from the corresponding sugar–amino acid direct reaction system.^17^ This is attributed to the asynchrony between glyoxal formation and methionine release during ARP degradation, as well as the rapid pH drop that inhibits the condensation of α-aminocarbonyl compounds.

### 2.3 Thermal self-condensation of hydroxy amino acids

Hydroxy amino acids (serine and threonine) can thermally generate pyrazines without the participation of sugars. Baltes and Bochmann found that heating serine or threonine alone could produce pyrazines, accounting for 32–38% of the total volatiles, confirming that hydroxy amino acids can directly generate α-aminoketone intermediates *via* pyrolysis (Scheme 4).^18^

**Scheme 4:**
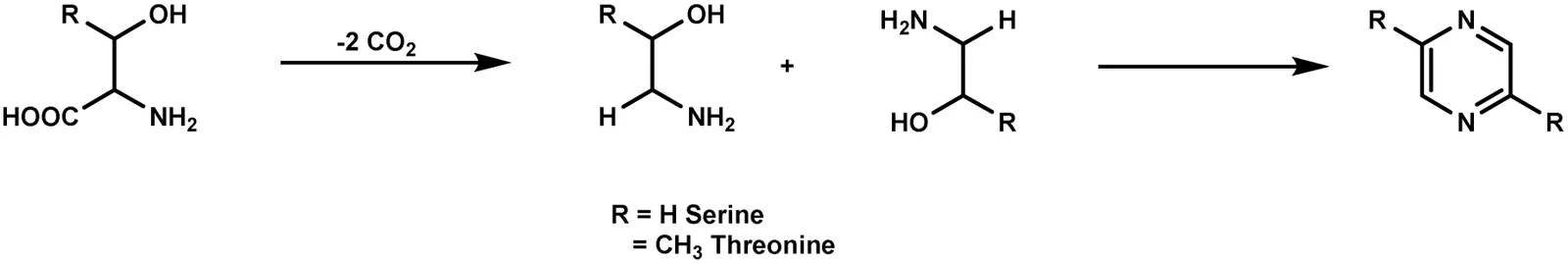
Decarboxylation mechanism proposed by Baltes and Bochmann.

Shu further investigated the pyrolysis products of l-serine and l-threonine, and found that serine mainly generated pyrazine, ethylpyrazine, and 2,6-diethylpyrazine, while threonine mainly generated 2,5-dimethylpyrazine, trimethylpyrazine, and 2-ethyl-3,6-dimethylpyrazine.^19^ Based on the product distribution, Shu revised the decarboxylation pathway proposed by Baltes and Bochmann, pointing out that decarbonylation followed by dehydration is the main route for the formation of α-aminocarbonyl intermediates, and that decarbonylation, rather than decarboxylation, is the key step in pyrazine formation from hydroxy amino acid pyrolysis (Scheme 5).^18^

**Scheme 5:**
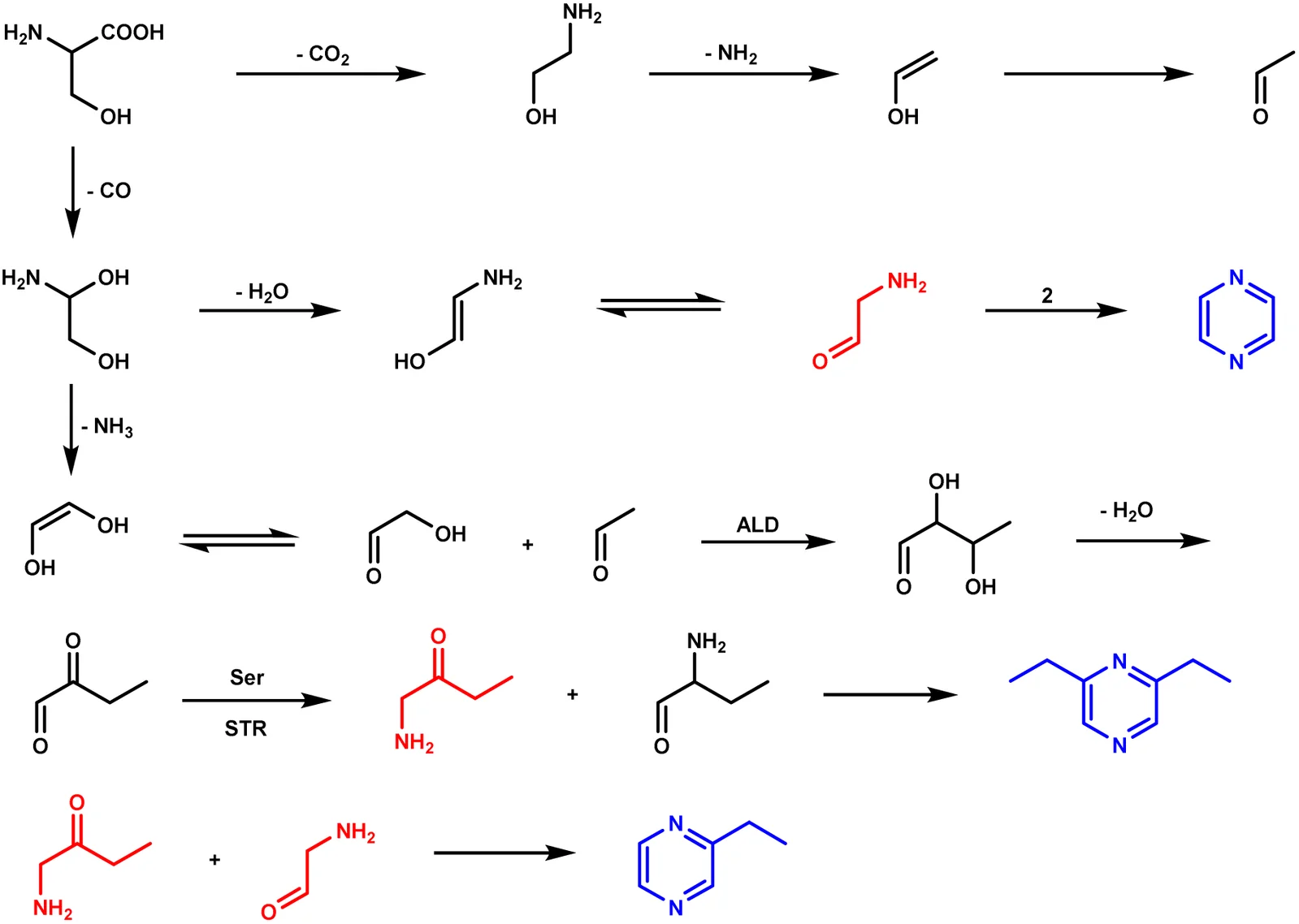
Decarbonylation mechanism proposed by Shu.

### 2.4 Involvement of lipid degradation products

Lipid oxidation products can also participate in the alkylation of the pyrazine ring. Huang *et al.* identified long-chain alkylpyrazines such as 2-methyl-3-pentylpyrazine and 2,5-dimethyl-3-pentylpyrazine in a zein–starch–corn oil model system, and proposed that pentanal generated from lipid oxidation condenses with dihydropyrazine intermediates, followed by dehydration and aromatisation to yield pentyl-substituted pyrazines.^20^ The addition of oil significantly increased the total pyrazine yield (Scheme 6).

**Scheme 6:**
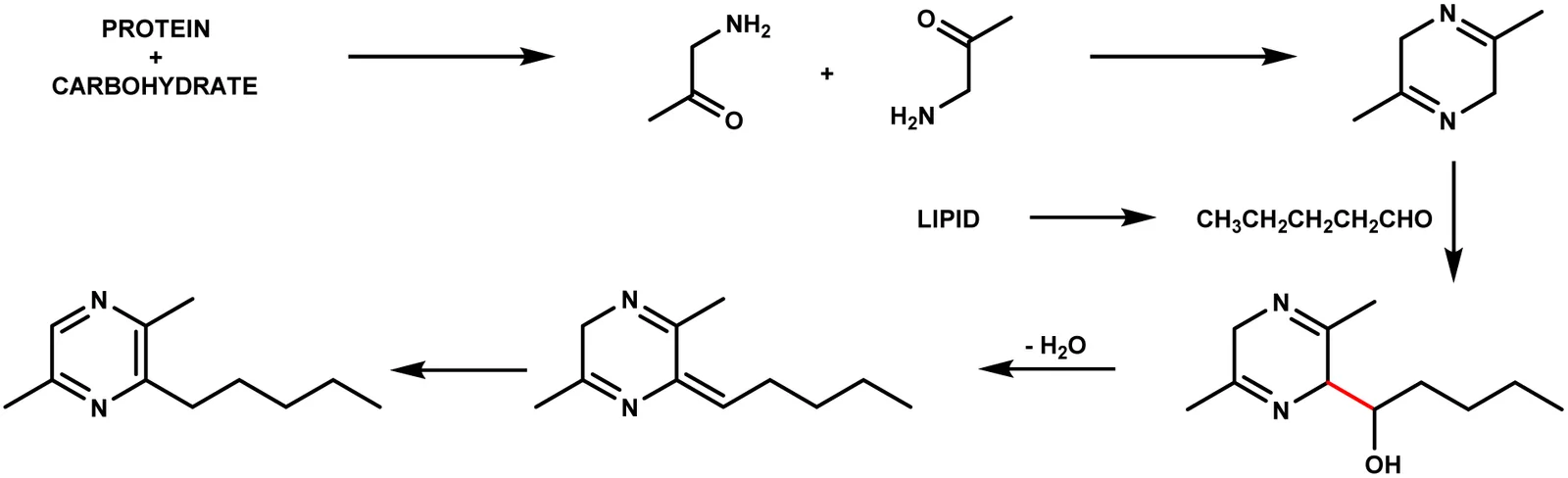
Mechanism of long-chain alkylpyrazine formation *via* condensation of lipid-derived aldehydes with dihydropyrazines.

Different oil types exhibited selective effects on the promotion of pyrazine homologues: Yu *et al.* found that olive oil was most favorable for the formation of 2,5-dimethylpyrazine, while sunflower oil was most favourable for 2,3,5-trimethylpyrazine;^21^ 2-propyl-3,6-dimethylpyrazine was detected only in flaxseed oil, suggesting that oxidation products of highly unsaturated oils can provide specific alkylation fragments for pyrazines. Hu *et al.* employed sequential enzymatic modification of sesame oil with lipase and lipoxygenase under mild conditions, and found that lipid carbonyl compounds generated by moderate enzymatic oxidation significantly promoted the formation of long-chain alkylpyrazines in Maillard model systems, offering a new approach for the controllable generation of lipid–Maillard synergistic flavours.^22^

### 2.5 Non-sugar precursor: ascorbic acid

Adams and De Kimpe first systematically investigated the pyrazine formation from ascorbic acid with 20 amino acids under dry-roasting conditions, identifying 36 alkylpyrazines, among which lysine exhibited the highest reactivity.^23^ Ascorbic acid degrades to generate α-dicarbonyl compounds such as methylglyoxal, glyoxal, and butanedione, which then form pyrazines through Strecker degradation and dihydropyrazine oxidation/alkylation pathways (Scheme 7). Due to the reducing nature of ascorbic acid, the oxidation of dihydropyrazine is inhibited, directing pyrazine formation more toward the alkylation pathway to generate 3-alkyl-substituted pyrazines, indicating that the redox properties of precursors exert an important regulatory role on the structure of pyrazine products.

**Scheme 7:**
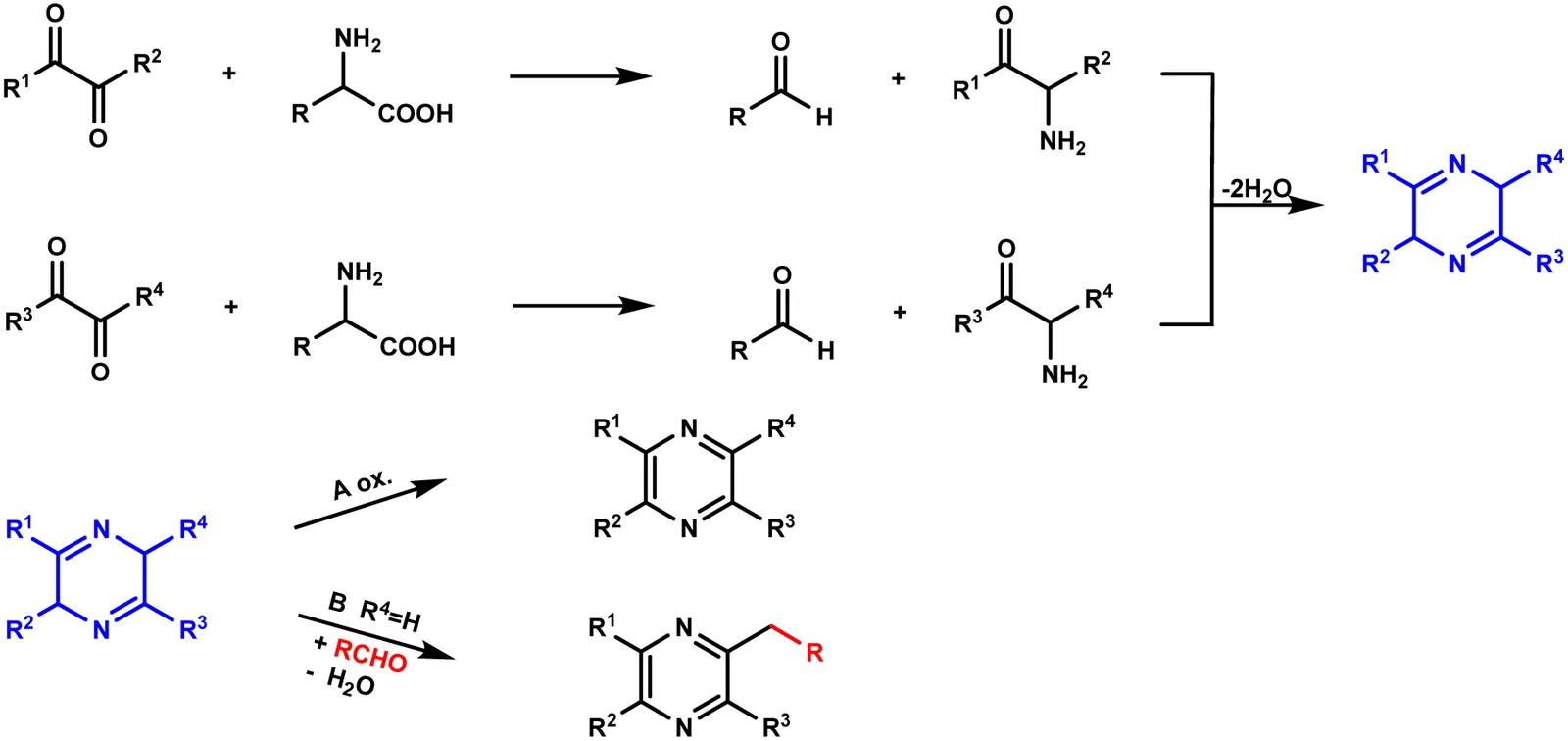
Proposed pathway for pyrazine formation from the reaction of ascorbic acid with amino acids (R, R^1^, R^2^ and R^3^ represent H or alkyl groups).

Pyrazine formation in the ascorbic acid–amino acid system is significantly regulated by pH. Under alkaline conditions, pyrazine production increased substantially, whereas under acidic conditions, only trace amounts of methylpyrazine were detected.^24^ The extent of pH influence varied with amino acid type—basic amino acids could generate pyrazines even at pH 5.0–6.0, exhibiting autocatalytic properties, while acidic amino acids were most affected by pH.^25^ This further confirms the differential regulation of pyrazine formation by the side-chain properties of amino acids.

### 2.6 Peptides and proteins as nitrogen sources

Free amino acids have long been considered the primary amino donors for pyrazine formation in the Maillard reaction. However, the content of peptides and proteins in food systems is far higher than that of free amino acids. Recent studies have shown that peptide-type nitrogen sources play a more significant role in pyrazine formation than free amino acids.

#### 2.6.1 Dipeptide/tripeptide model systems and *N*-terminal amino acid effects

Van Lancker *et al.* found that lysine-containing dipeptide systems produced significantly higher total pyrazine yields than the corresponding free amino acid mixtures.^26,27^ Furthermore, the *N*-terminal amino acid structure of the dipeptide was found to exert a decisive influence on pyrazine yield: when the *N*-terminus was glycine, alanine, or serine, pyrazine production was relatively high, whereas when it was proline, valine, or leucine, pyrazine yield was extremely low. Wang *et al.* further confirmed that dipeptides outperformed tripeptides, and that the dominant role of the *N*-terminal amino acid was also observed in tripeptide systems.^28^

#### 2.6.2 Pyrazine-forming ability of protein hydrolysates

Scalone *et al.* confirmed that peptide-type nitrogen sources contribute far more to pyrazine formation than free amino acids, and this pattern was also validated in plant protein systems.^29–33^ The pyrazine-forming ability of protein hydrolysates from different sources followed the order: animal-derived (chicken bone) > plant-derived (wheat > rice), which was consistent with the consumption rate of <1 kDa small-molecule peptides, indicating that low-molecular-weight peptide fractions are the key active components.^32^ Nishimura and Abe further identified that cysteine-containing peptide sequences were preferentially consumed during the Maillard reaction, suggesting that specific peptide sequences serve as key precursors for pyrazines.^33^

#### 2.6.3 Peptide-derived special intermediates and products

Zou *et al.* employed LC-MS to identify, for the first time, peptide–Amadori conjugates, providing direct evidence at the intermediate level that peptides preferentially participate in the Maillard reaction.^34^ Sulfur-containing oligopeptides also exhibit pyrazine-forming activity: Zou *et al.* found that Cys–Gly–Val displayed the highest activity, whereas Val–Met remained essentially inert.^35^ CAMOLA isotope labelling experiments further revealed that, in the Leu–Cys system, xylose contributed only two carbon atoms to the pyrazine ring of 2-isobutylpyrazine, with the remaining carbons derived from the leucine carbon skeleton (Scheme 8). Scalone *et al.* validated the promoting effect of whey protein hydrolysates on the formation of 2,5-dimethylpyrazine in biscuits and bread, demonstrating that moderately hydrolysed peptides represent an ideal class of pyrazine precursors.^36^ In addition, no pyrazines were detected in the reaction of a pentapeptide with sugars, suggesting that excessively long peptide chains may lose pyrazine-forming activity due to the lack of free amino groups generated upon thermal cleavage.^37^

**Scheme 8:**
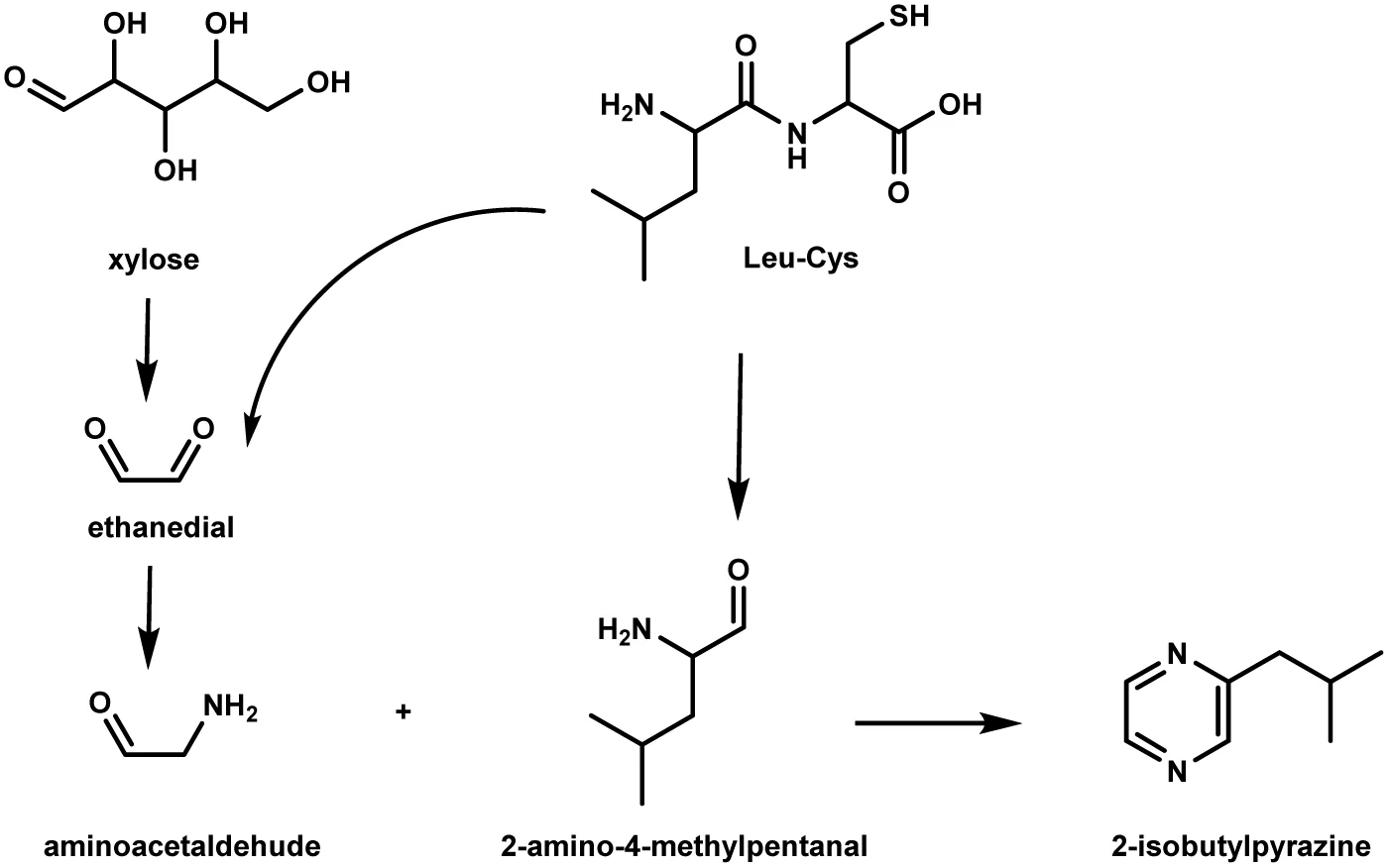
Proposed formation pathway of 2-isobutylpyrazine in the peptide–xylose Maillard reaction.

## 3. Chemical mechanisms of pyrazine formation

The construction of the pyrazine ring involves multiple chemical pathways, and the understanding of its molecular mechanisms has undergone continuous refinement over the course of research. Moreover, the alkyl substituents on the pyrazine ring can undergo further secondary reactions, extending the mechanistic picture of pyrazine formation from initial ring construction to subsequent side-chain modification.

### 3.1 Classical α-aminoketone condensation–oxidation pathway

Newell *et al.* proposed the earliest mechanistic hypothesis for pyrazine formation: amino acids react with reducing sugars *via* Amadori rearrangement and Strecker degradation to generate α-aminoketones, two molecules of which undergo self-condensation to form dihydropyrazine, followed by oxidation to yield pyrazine (Scheme 9).^38^

**Scheme 9:**
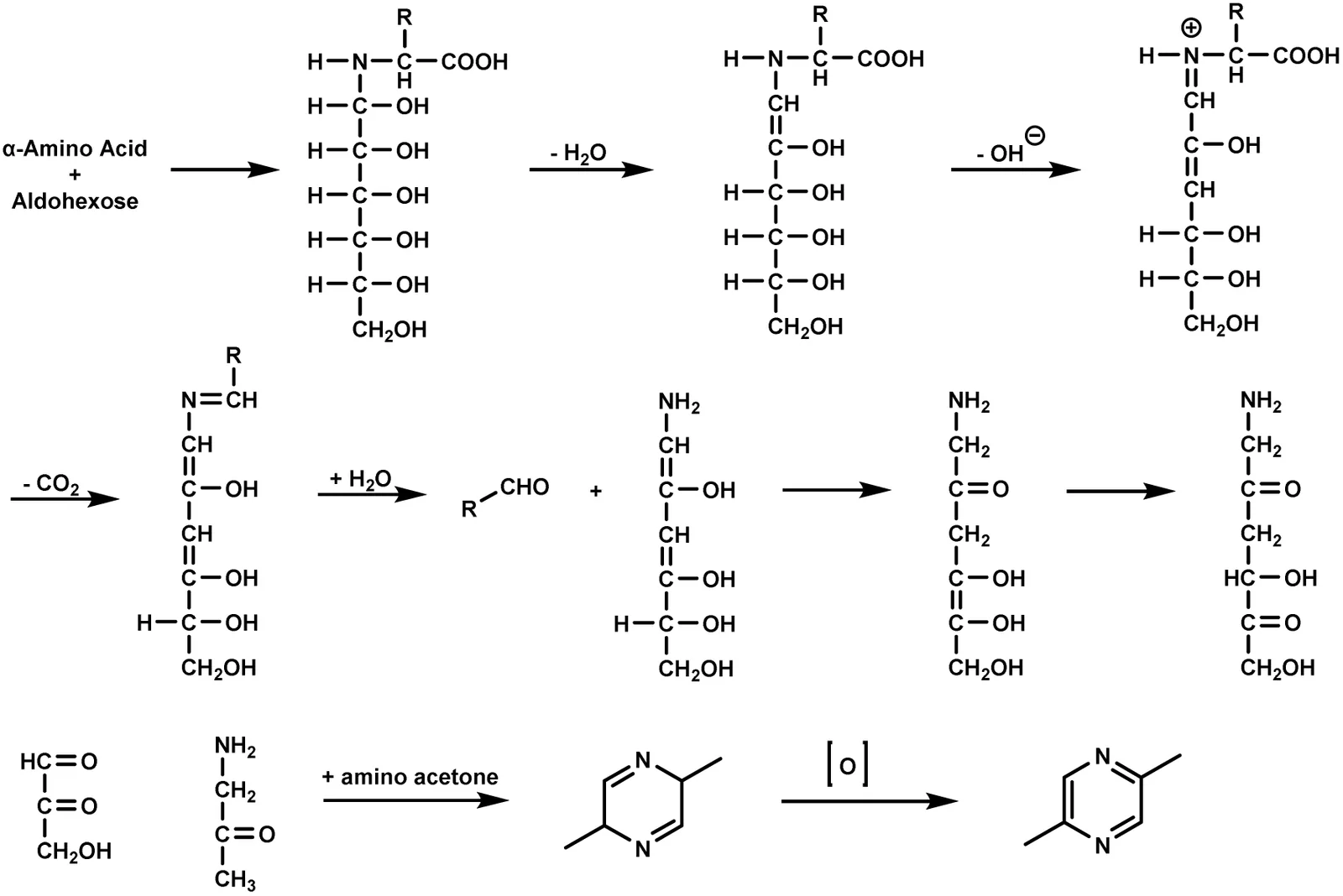
Postulated mechanism for conversion of amino acids and sugars to pyrazine (R represent H or alkyl groups).

Because Strecker degradation is the key step in this pathway, it is shown separately in Scheme 10. The α-amino acid first condenses with an α-dicarbonyl compound to form a Schiff base, which undergoes decarboxylation to give an imine intermediate; subsequent hydrolysis releases a Strecker aldehyde (R–CHO) and an α-aminoketone. The α-aminoketone then participates in the self-condensation–oxidation sequence leading to pyrazine formation.

**Scheme 10:**
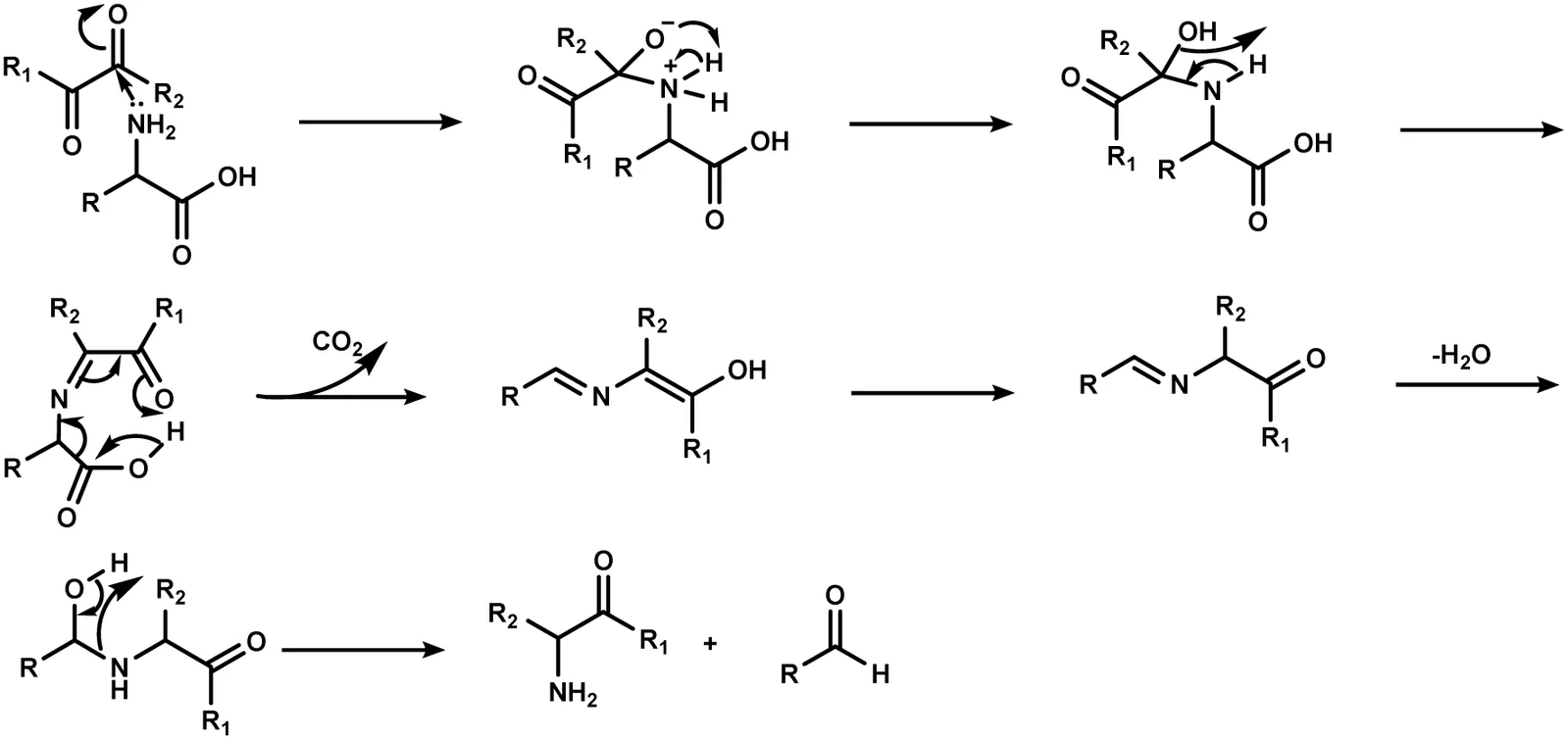
Mechanism of Strecker degradation (R represents the side chain of the α-amino acid; R^1^ and R^2^ represent H or alkyl groups derived from the α-dicarbonyl compound).

Shibamoto and Bernhard further proposed ten α-aminocarbonyl intermediates, and pointed out that the manner of sugar fragmentation determines the relative concentrations of these intermediates, thereby influencing the distribution of pyrazines.^12^ Recent computational chemistry studies have validated the feasibility of this pathway at the molecular level.^39^ This pathway represents the most common mode of pyrazine formation in sugar–amino acid systems, and the subsequently discovered radical and azomethine ylide pathways serve as supplements and extensions to this fundamental framework.

### 3.2 Isotopic labelling verification of the Strecker degradation pathway

Amrani-Hemaimi *et al.* demonstrated through ^13^C isotope labelling experiments that carbon atoms from amino acids participate in the construction of pyrazine side chains.^40^ By reacting [3-^13^C] alanine and [2-^13^C] glycine with glucose, respectively, it was found that the C3 atom of alanine was selectively incorporated into the ethyl C2 position of ethylpyrazines, while the C2 atom of glycine was incorporated into one of the methyl groups of trimethylpyrazine. These results indicate that amino acids serve not only as a nitrogen source, but also contribute to the formation of substituents on the pyrazine ring *via* their Strecker aldehyde degradation products. The same labelling strategy has also been extended to [3-^13^C] alanine, which generates ^13^C-labelled acetaldehyde and is incorporated into the ethyl group of ethylpyrazine (Scheme 11).

**Scheme 11:**
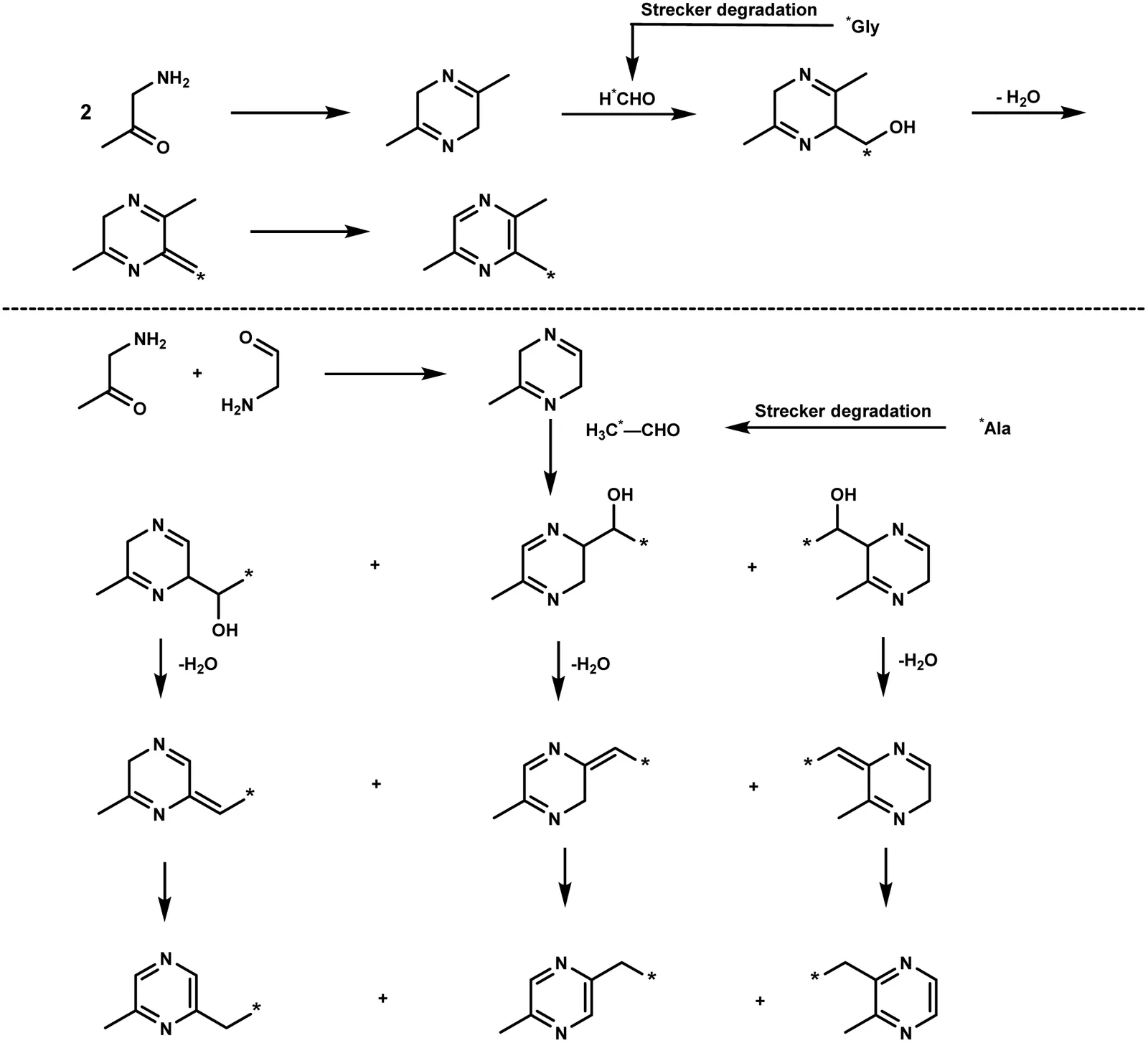
Formation of pyrazines from isotopically labelled amino acids. [2-^13^C] Glycine generates ^13^C-labelled formaldehyde, which is incorporated into the methyl groups of trimethylpyrazine; [3-^13^C] Alanine generates ^13^C-labelled acetaldehyde, which is incorporated into the ethyl group of ethylpyrazine.

### 3.3 Dehydration *vs.* dehydrogenation in aromatisation

Dihydropyrazine must undergo aromatisation to form a stable pyrazine ring. The conventional view holds that this process proceeds *via* oxidative dehydrogenation. However, Shibamoto and Bernhard observed that pyrazine formation in a glucose–ammonia system does not depend on oxygen, and proposed that aromatisation may proceed through dehydration of hydroxy dihydropyrazine in certain systems.^41^ DFT calculations by Shipar demonstrated that oxidation remains necessary for the conversion of non-hydroxylated dihydropyrazine to pyrazine.^42^ Thus, the aromatisation pathway depends on the structure of the intermediate: hydroxy dihydropyrazine undergoes aromatisation *via* dehydration, whereas non-hydroxylated dihydropyrazine proceeds *via* oxidation, with the two pathways coexisting under different conditions (Scheme 12).

**Scheme 12:**
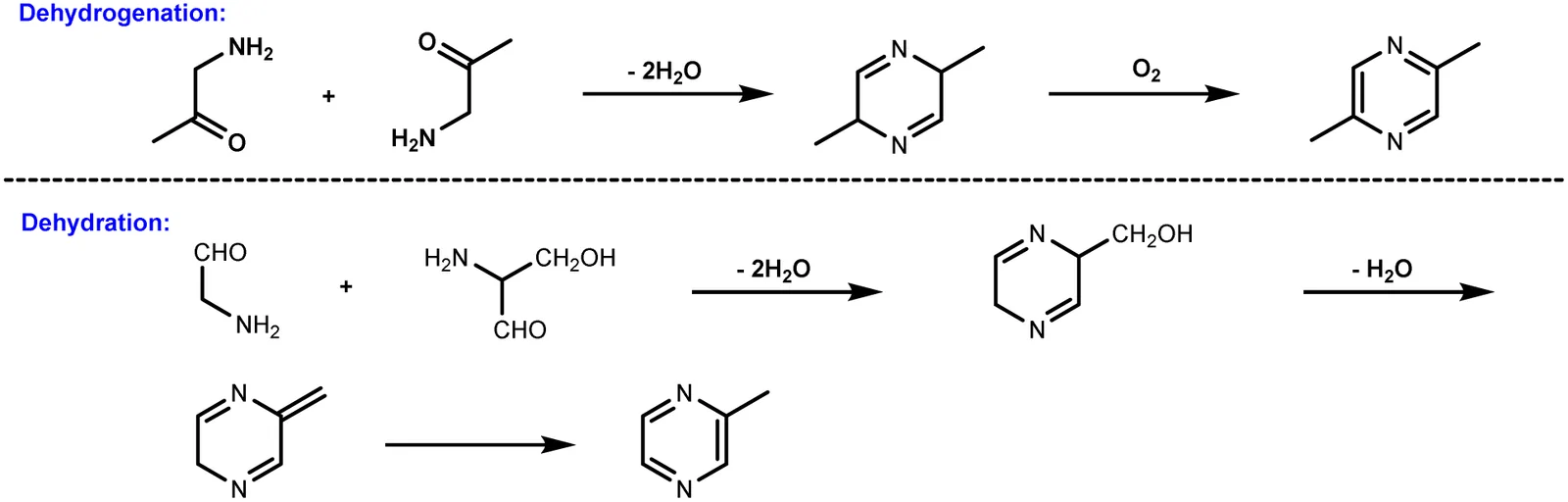
Dehydration *vs.* dehydrogenation pathways for the aromatisation of dihydropyrazine to pyrazine.

### 3.4 Radical mechanism

Hayashi and Namiki first confirmed the formation of 1,4-dialkylpyrazine cation radicals in the reaction of d-glucose with butylamine by ESR spectroscopy, and proposed a radical pathway for pyrazine formation: following the condensation of sugar and amine to form a Schiff base, subsequent C–C cleavage, cyclisation, oxidation and reduction steps lead to the formation of pyrazine.^43^ This work was the first to link the ring construction of pyrazines with radical chemistry. Milić and Piletić verified the same radical species in a glucose–aminobutyric acid system, and found that this system could generate pyrazines bearing long-chain substituents, indicating that the products of the radical pathway are not limited to alkylpyrazines (Scheme 13).^44^

**Scheme 13:**
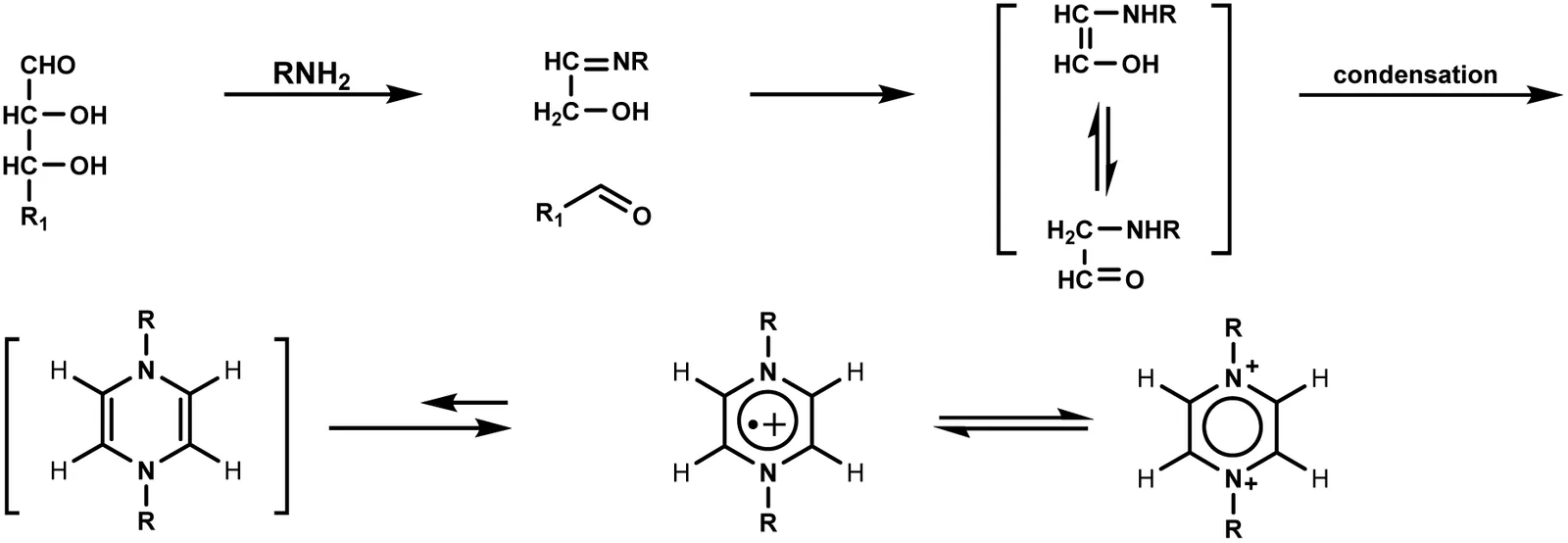
Proposed pathway for the radical mechanism of pyrazine formation (R represents an alkyl or aryl group).

### 3.5 Azomethine ylide dimerisation pathway

Guerra and Yaylayan demonstrated a pyrazine formation pathway that does not depend on α-dicarbonyl compounds using a glyoxylic acid–glycine model system.^45^ Condensation of glycine with glyoxylic acid forms a Schiff base, which undergoes cyclisation and decarboxylation to generate an azomethine ylide. Subsequent dimerisation, followed by oxidative decarboxylation and dehydrogenation, leads to pyrazine formation. This pathway proceeds efficiently even in the presence of α-dicarbonyl compounds, indicating that it is not a minor side route but rather an independent channel operating in parallel with the Strecker pathway. Thus, the mechanistic understanding of pyrazine formation has expanded from being solely dependent on α-dicarbonyl compounds to encompassing multiple types of precursors (Scheme 14).

**Scheme 14:**
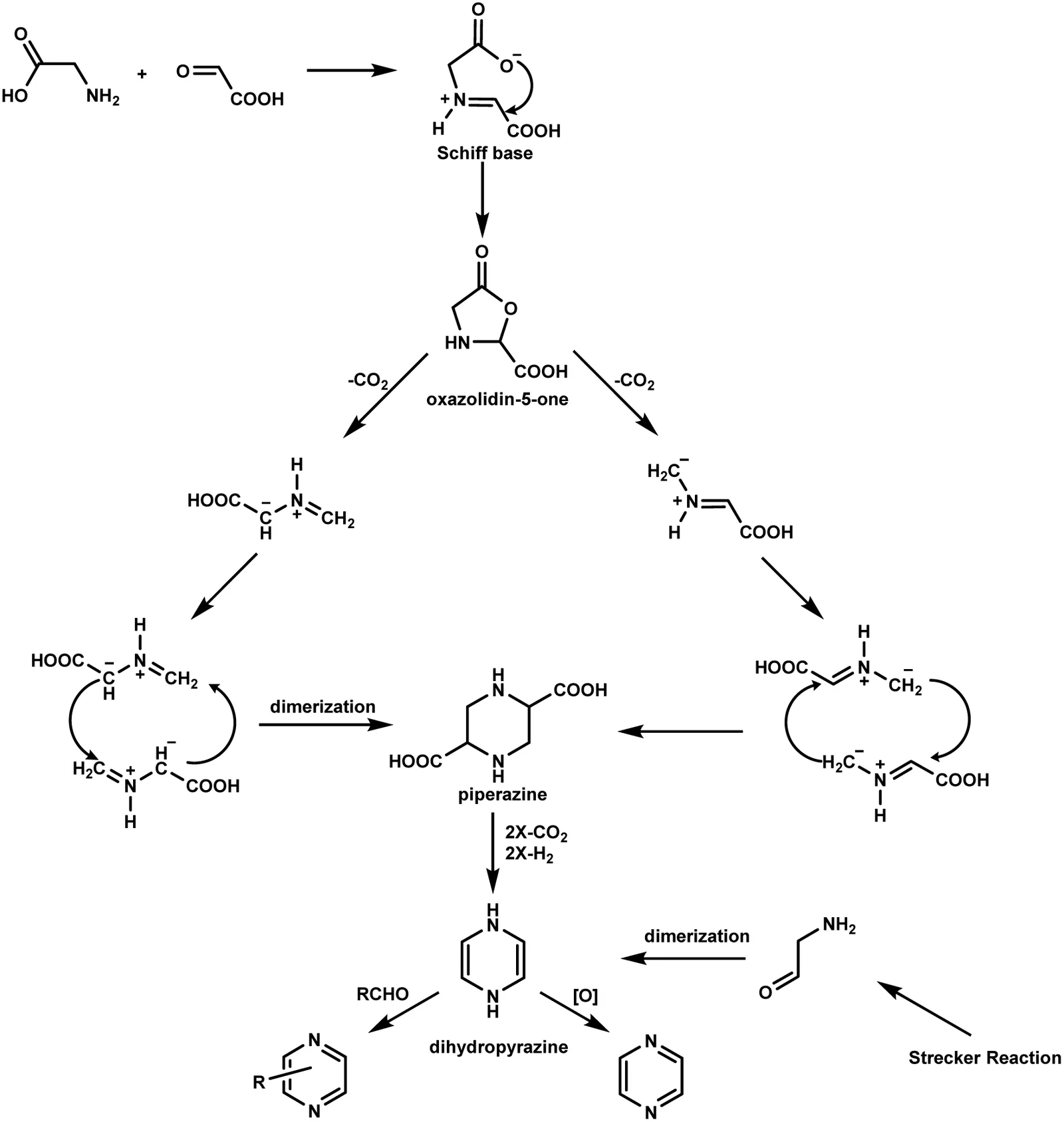
Generation and dimerisation of azomethine ylides from glycine and glyoxylic acid.

### 3.6 Special mechanism involving peptides

Peptides lack a free carboxyl group and therefore cannot undergo classical Strecker degradation. Van Lancker *et al.* proposed an alternative mechanism for the formation of α-aminoketones from peptides: condensation of a dipeptide with an α-dicarbonyl compound forms an imine, which undergoes deprotonation, 1,5-H migration, enolisation and hydrolysis to generate an α-aminoketone, along with the release of a complex α-dicarbonyl compound.^27^ This pathway does not release Strecker aldehydes, thereby reducing side reactions and resulting in a higher proportion of pyrazines in the total volatile fraction (Scheme 15). Zou *et al.* identified peptide–Amadori conjugates for the first time in a peptide–xylose reaction by LC-MS, confirming this mechanism at the intermediate level.^34^ Compared with the free amino acid pathway, the peptide pathway does not require decarboxylation and does not produce Strecker aldehydes, leading to fewer side reactions and higher selectivity towards pyrazines. This provides a mechanistic explanation for the experimental observations described in Section 2, that peptides are more efficient pyrazine precursors than free amino acids.

**Scheme 15:**
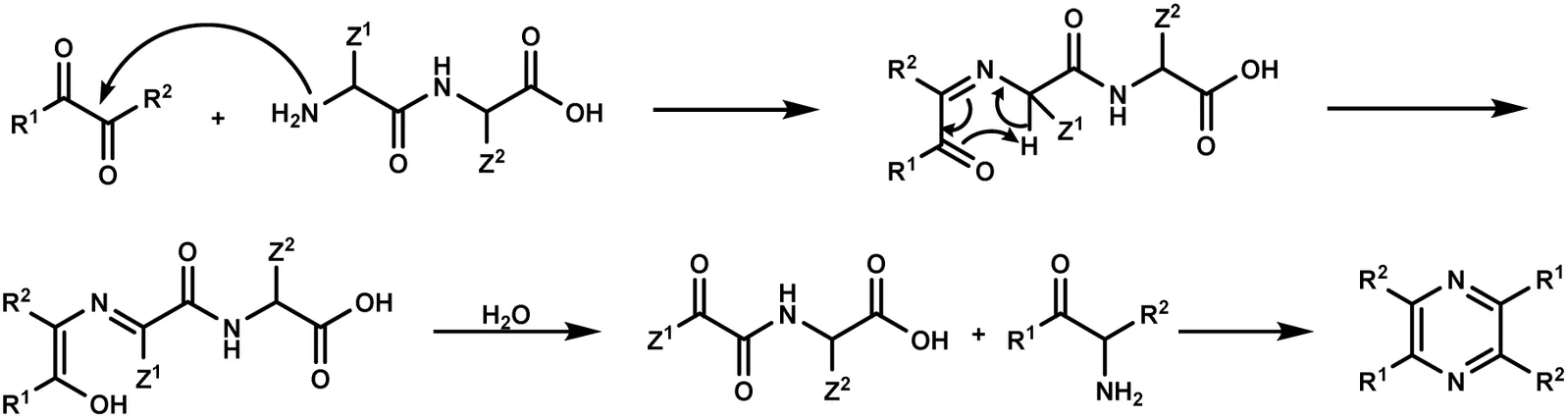
Proposed mechanism for pyrazine formation from peptides (R^1^ and R^2^ represent H or alkyl groups; Z^1^ and Z^2^ represent the side chains of the amino acid residues in the peptide).

### 3.7 Formation mechanism of vinylpyrazines

The alkyl substituents on the pyrazine ring can undergo further secondary reactions. Ma *et al.* elucidated the formation mechanism of 2-vinylpyrazine and 2-vinyl-6-methylpyrazine.^46^^13^C isotope labelling revealed that the pyrazine ring carbons originate from glucose, while the vinyl carbons are derived from formaldehyde. Model reactions confirmed that alkylpyrazines can condense directly with formaldehyde to form vinylpyrazines. H/D exchange experiments identified the methyl C–H bond as the reactive site. On the basis of this evidence, the study proposed that the methyl group of alkylpyrazines undergoes enolisation, followed by aldol condensation with formaldehyde and subsequent dehydration to yield vinylpyrazines. This extends the mechanistic picture of pyrazine formation from initial ring construction to side-chain secondary modification (Scheme 16).

**Scheme 16:**
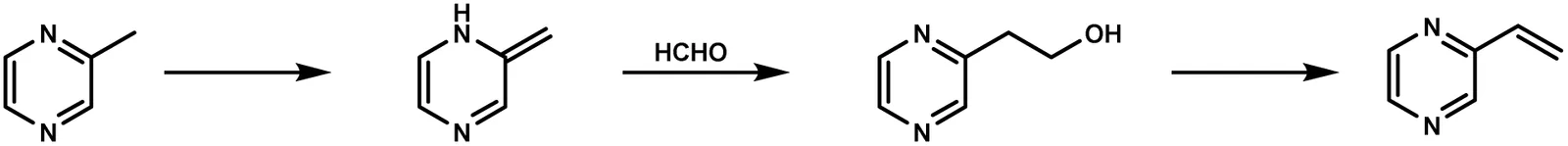
Proposed mechanism for the formation of vinylpyrazines.

In addition, the dimerisation–electrophilic capture of α-aminoketones provides a synthetic chemical route for the construction of the pyrazine ring. González-Rodríguez *et al.* reported the mechanism of the reaction of aminoacetone with electrophiles such as aldehydes and alkynes in phosphate buffer to generate trisubstituted unsymmetrical pyrazines, which can be coupled with l-threonine dehydrogenase to achieve a biocatalytic cascade synthesis (Scheme 17).^47^

**Scheme 17:**
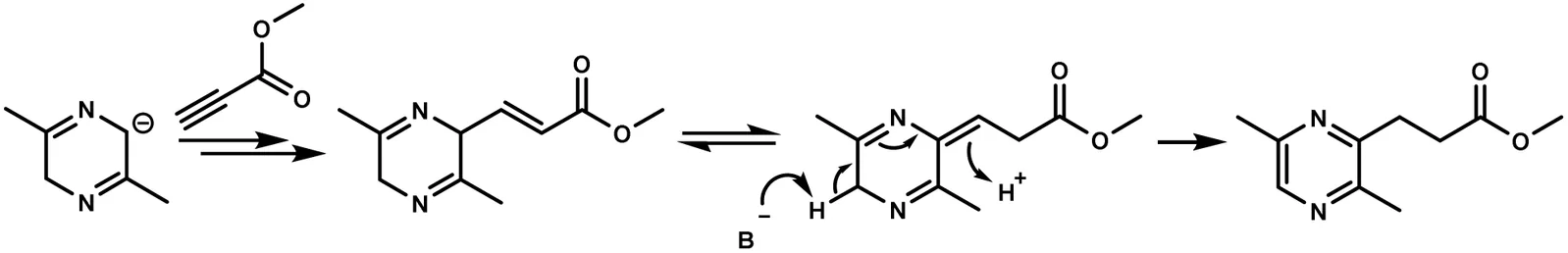
Dihydropyrazine addition to alkynes for the formation of unsymmetrical pyrazines.

### 3.8 Formation of pyrazinones

Pyrazinones are a class of oxygen- and nitrogen-containing heterocyclic compounds structurally related to pyrazines, formed in the Maillard reaction. Luo *et al.* first identified eight pyrazinones in the reactions of glycylalanine and alanylglycine dipeptides with α-dicarbonyl compounds, and elucidated their formation pathways involving dehydration, tautomerisation and decarboxylation (Scheme 18).^48^ Sensory evaluation revealed for the first time the aroma characteristics of pyrazinones: 1-ethyl-2(1*H*)-pyrazinone exhibited cereal/biscuit-like notes, while 1,3-dimethyl-2(1*H*)-pyrazinone displayed green/herbaceous notes. The structural differences between pyrazinones and pyrazines lead to distinctly different aroma profiles, indicating that oxidative modification of the pyrazine ring in the Maillard reaction can generate compound classes with entirely different flavour characteristics, thereby expanding the research boundaries of pyrazine flavour chemistry.

**Scheme 18:**
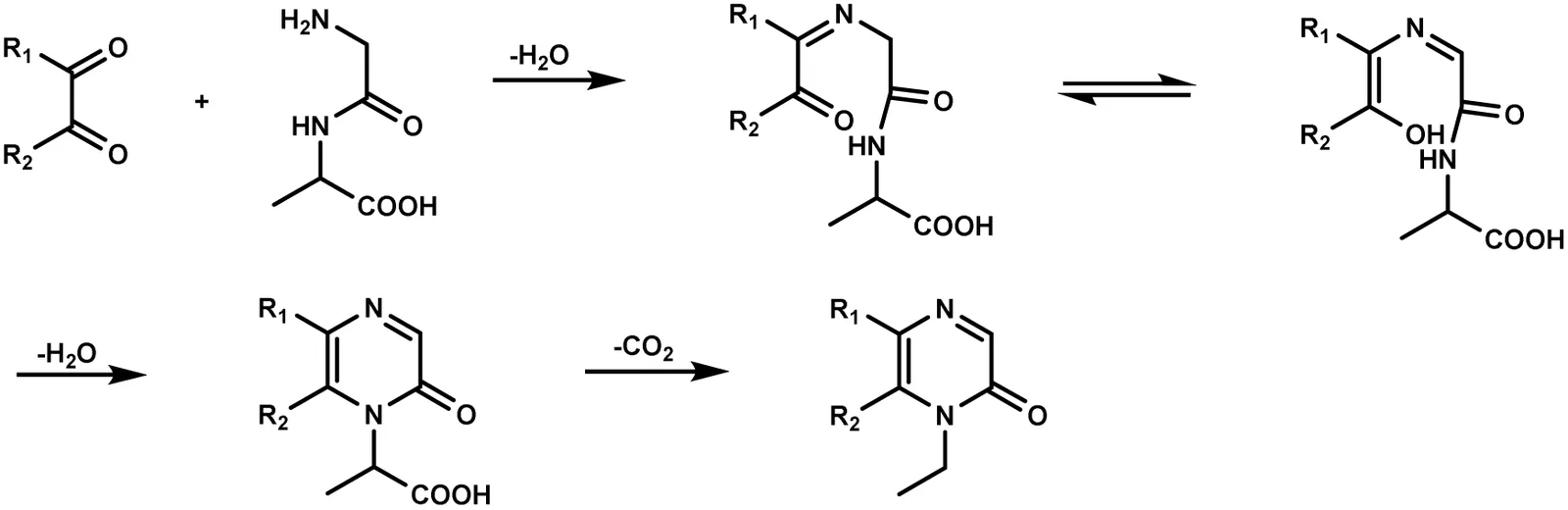
Proposed formation pathways of pyrazinones (R^1^ and R^2^ represent H or alkyl groups).

## 4. Factors influencing pyrazine formation and strategies for regulation

The yield and distribution of pyrazines are governed by multiple factors, and understanding the underlying principles is essential for achieving targeted synthesis.

### 4.1 Temperature and time

Koehler and Odell found that in the range of 80–150 °C, virtually no pyrazines were generated below 100 °C; between 100 and 150 °C, the yield increased rapidly with rising temperature, while above 150 °C, the yield fluctuated.^10^ At 120 °C, the yield increased rapidly within 24 h and then plateaued.

Lin *et al.* found that dimethylpyrazines and ethylpyrazine followed first-order kinetics, whereas trimethylpyrazine, tetramethylpyrazine and ethyl–methylpyrazines conformed to a zero-order model, among which 2-ethyl-5-methylpyrazine exhibited the lowest apparent activation energy.^49^ Gao *et al.* constructed a temperature-dependent formation profile for 15 pyrazines in the range of 60–180 °C using an Amadori compound pyrolysis model, with most alkylpyrazines reaching their maximum yields at 140 °C (see Section 5.2 for details).^39^ Taken together, the effect of temperature on pyrazine formation is dual in nature: appropriate temperature elevation accelerates the reaction, but excessively high temperatures may lead to decomposition or volatilisation losses of pyrazines, indicating the existence of an optimal temperature window.

### 4.2 pH and base catalysis

Koehler and Odell found that the addition of base significantly promoted pyrazine formation, while the addition of acid almost completely suppressed it.^10^ DFT calculations by Jalbout and Shipar independently confirmed, from a thermodynamic perspective, that alkaline conditions are the most favourable.^42,50^

Yu *et al.* confirmed in an ascorbic acid–amino acid system that alkaline conditions (pH 8.0–9.0) favour pyrazine formation, although the degree of influence varies with amino acid type – acidic amino acids are most strongly affected by pH, while basic amino acids are least affected, and can even generate pyrazines at pH 5.0–6.0.^25^ Glutamic acid represents a notable exception to this trend: despite being an acidic amino acid, it effectively promotes pyrazine formation under near-neutral conditions. Zhan *et al.* revealed through DFT calculations that deprotonation of the γ-carboxyl group (p*K*_a_ = 4.29) can remotely activate the nucleophilicity of the amino group.^51^ This finding challenges the conventional view that acidic amino acids exhibit low reactivity under near-neutral conditions, demonstrating that the spatial arrangement and electronic effects of amino acid side-chain functional groups can override the intrinsic acid–base properties of the α-amino group, exerting a decisive influence on pyrazine formation. Hua, Chu *et al.* verified the promoting effect of alkaline conditions in a lysine–glucose system, with higher lysine proportions leading to more pronounced pyrazine formation.^52,53^

### 4.3 Precursor structure and nitrogen source type

Wong and Bernhard found that different nitrogen sources profoundly influence the type and yield of pyrazines, refuting the hypothesis that “free ammonia is the sole nitrogen intermediate”.^54^ Koehler and Odell further pointed out that the products derived from inorganic nitrogen sources are almost exclusively unsubstituted pyrazines, demonstrating that the alkyl substituents mainly originate from the carbon skeleton of amino acids.^10^ Hwang *et al.*, using ^15^N isotope labelling, revealed the synergistic and competitive relationships in multi-nitrogen-source systems: lysine can synergistically enhance the pyrazine-forming ability of glycine, while arginine exhibits an inhibitory effect.^55^

Regarding the influence of peptide-type nitrogen sources, the core conclusions have been discussed in detail in Section 2.6 and are not repeated here. In addition, Xiao *et al.* found that proline-specific compounds were present at much higher levels than pyrazines in an asparagine–proline–glucose system, indicating that the reaction of proline with α-dicarbonyl compounds has a lower activation energy and thus outcompetes other amino acids.^56^

### 4.4 Reaction medium and emulsion systems

Yu *et al.* found that 50% (v/v) ethanol–water medium was the most favourable for pyrazine formation.^57^ Troise *et al.* observed that the more hydrophobic leucine exhibited a 47% reduction in Amadori product formation in emulsion systems, attributed to its anchoring at the oil–water interface.^58^ In heterogeneous systems, a portion of pyrazines may partition into the oil phase, reducing the product concentration in the aqueous phase through an extraction–migration effect, thereby shifting the reaction equilibrium.^21^

Xia *et al.* found that the fluorescence intensity in a peptide–glucose Maillard reaction first increased and then decreased, in parallel with browning, which can serve as an indicator for monitoring the reaction progress and provide a reference for the precise regulation of pyrazine formation.^59^ In addition, the gas atmosphere of the reaction system also influences pyrazine formation. Yuan *et al.* found that high oxygen concentrations increased the total pyrazine yield by 44% in a methionine–glucose system, attributed to the promotion of α-dicarbonyl compound formation and their subsequent conversion to pyrazines under high oxygen conditions.^60^ Collectively, these studies demonstrate that the influence of reaction medium on pyrazine formation is no less significant than the traditionally emphasised factors of temperature and pH, representing an underexploited dimension in the targeted synthesis of pyrazines.

### 4.5 Maillard reaction and pyrazine formation in an ultrasonic field

The cavitation effect of high-intensity ultrasound generates local high temperatures and transient high pressures, promoting the Maillard reaction. Yu *et al.* found that ultrasound treatment significantly reduced the activation energies for the formation of key intermediates: 1-deoxyosone decreased from 105.6 to 60.9 kJ mol^−1^, and 3-deoxyosone from 89.5 to 51.4 kJ mol^−1^.^61^ The *E*_a_ values for 2,5-dimethylpyrazine, 2,3,5-trimethylpyrazine, 3-ethyl-2,5-dimethylpyrazine and 2,3,5,6-tetramethylpyrazine were reduced by approximately 11%, 24%, 26% and 20%, respectively. In addition, 3,5-dimethyl-2-vinylpyrazine and 3,5-diethyl-2-methylpyrazine were detected only in the ultrasound-treated samples.

### 4.6 Inhibitory factors in pyrazine formation

Phenolic compounds can inhibit pyrazine formation by scavenging Maillard reaction intermediates. Totlani and Peterson found that epicatechin reduced the yields of various pyrazines in a glucose–glycine system by dozens to hundreds of times, and isotope labelling confirmed that it directly captures C2, C3 and C4 reactive fragments generated from sugar degradation.^62^ In bread models, rosmarinic acid and epigallocatechin gallate also exhibited pyrazine-inhibitory effects.^63^

Wang *et al.* found that ellagic acid significantly reduced pyrazine yields when added during the accumulation phase of Amadori compounds, with the inhibitory effect increasing with pH.^64^ Ellagic acid can be hydrolysed to an anion that forms adducts with Amadori compounds, delaying their degradation. However, the effect of ellagic acid is dependent on the timing of addition: when added during the accumulation phase of Amadori compounds, it inhibits pyrazine formation; when added at the initial stage of the reaction, ellagic acid has not yet encountered capturable intermediates, and its oxidation products instead promote the aromatisation of dihydropyrazines, leading to increased pyrazine yields. l-cysteine also inhibits browning by reducing the formation of deoxyosones and glyoxal.^65^

In addition, high-pressure treatment (600 MPa, 60 °C) can reduce the total pyrazine yield to approximately 3% of that under atmospheric thermal treatment, providing a reference for the inhibition of pyrazines in low-temperature processing scenarios.^66^ Overall, strategies for pyrazine inhibition can be classified into two categories: chemical inhibition and physical inhibition. The former is applicable to formulation regulation, while the latter is suitable for process optimisation, and the two approaches can be flexibly employed or combined according to specific requirements.

## 5. Application of computational chemistry in the study of pyrazine formation mechanisms

Maillard reaction intermediates are highly reactive, short-lived and present at low concentrations, making them difficult to capture directly by experimental means. Computational chemistry methods, particularly density functional theory (DFT), provide an effective means of elucidating the formation pathways and thermodynamic driving forces of pyrazines at the molecular level. Compared with experimental approaches, DFT can observe the intermediates and transition states of each elementary step in a reaction pathway, offering a molecular-level theoretical explanation for macroscopically observed phenomena. In recent years, research has expanded from thermodynamic calculations of single elementary reactions to kinetic analyses of complete reaction networks.

### 5.1 Thermodynamic feasibility studies of pyrazine formation

Shipar employed a dihydroxyacetone–glycine model system to investigate the formation pathways of 2,5-dimethylpyrazine under different pH conditions.^42^ Thermodynamic analysis revealed that the feasibility of 2,5-dimethylpyrazine formation followed the order: alkaline > neutral > isoelectric point > acidic. Jalbout and Shipar obtained the same pH dependence in a hydroxyacetaldehyde system.^50^ Both studies also indicated that Strecker degradation products are the most likely precursors for pyrazine ring formation, suggesting that Strecker degradation–dimerisation–oxidation represents a general pathway for pyrazine formation. However, these calculations were based on the Gibbs free energy differences (Δ*G*°) between products and reactants, and did not locate transition states, thus failing to provide direct information on activation energy barriers and kinetic rates. Thermodynamic feasibility only answers whether a reaction can occur, but cannot answer how fast it proceeds or which step is rate-limiting. Such kinetic questions require transition state calculations of complete reaction networks to be addressed. In recent years, the application of DFT methods has expanded from thermodynamic analysis to kinetic simulations of full reaction networks.

### 5.2 DFT studies on complete reaction networks

Gao *et al.* investigated the full reaction pathway for the formation of 3-ethyl-2,5-dimethylpyrazine from the pyrolysis of an alanine–glucose Amadori compound.^39^ Energy barrier calculations revealed that the cyclisation–aromatisation of the dihydropyrazine intermediate is the highest energy barrier step in the overall pathway, constituting the key rate-limiting step (Scheme 19). The overall energy barriers for the pyrolysis of ARPs under acidic conditions were significantly lower than those under alkaline pathways.

**Scheme 19:**
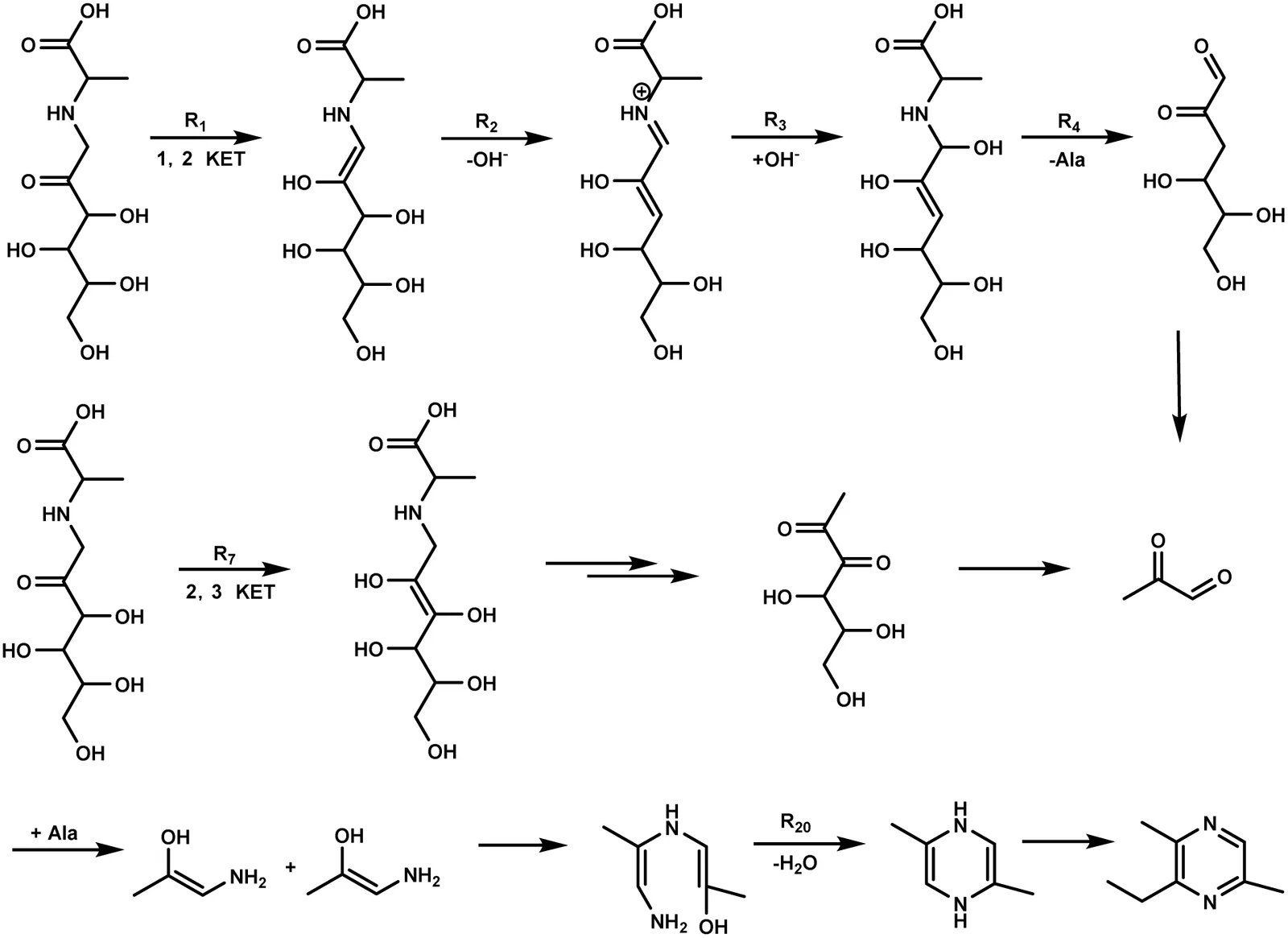
Energy profile for the formation of 3-ethyl-2,5-dimethylpyrazine from Amadori compound pyrolysis (R denotes the sequential reaction step).

Experimentally, pyrolysis of ARPs produced 25 volatile compounds, of which 15 were pyrazines, confirming that Amadori compounds are key precursors for pyrazine formation. 2,5-Dimethylpyrazine was detectable at 60 °C, while most alkylpyrazines reached their maximum yields at 140 °C. Zhan *et al.* revealed at the electronic structure level that deprotonation of the γ-carboxyl group of glutamic acid (p*K*_a_ = 4.29) raised the HOMO energy level by 4.92 eV, thereby enhancing the nucleophilic attack ability of the amino group.^51^ If Gao *et al.* answered the question of “which step is the slowest in the reaction pathway”, then Zhan *et al.* addressed “why certain amino acids exhibit higher reactivity at specific pH values”.^39,51^ Together, these two studies provide a theoretical interpretation of pyrazine formation mechanisms from the perspectives of reaction kinetics and electronic structure, respectively.

## 6. Conclusions and outlook

This review has systematically examined the research progress in the biomimetic and Maillard route synthesis of pyrazines since the 1960s. From three main perspectives – precursor types, formation mechanisms and regulatory strategies – the following core understandings can be drawn.

The precursors for the biomimetic synthesis of pyrazines have evolved from sugar–amino acid/ammonia model systems, on the one hand simplified to small carbonyl compounds, and on the other hand expanded to hydroxy amino acids, ascorbic acid and peptides/proteins, while lipid degradation products have been found to serve as alkylating agents participating in the modification of the pyrazine ring. This evolution reflects a shift in research perspective from complete sugar–amino acid reactions towards condensation of key fragments, as well as a continuous extension towards multi-component systems that more closely resemble real foods. Small-molecule carbonyl compounds can undergo direct condensation with high yields and good selectivity, while sugar–amino acid/ammonia model systems allow the pyrazine distribution to be tuned by selecting sugars with different structures. Hydroxy amino acids provide a sugar-independent pyrolysis pathway, and ascorbic acid can serve as a non-sugar precursor under relatively mild conditions. Among these, 6-deoxy sugars (*e.g.*, rhamnose) exhibit a yield approximately ten times that of hexoses, as the C-6 methyl group can participate in ethyl introduction without cleavage; peptides and proteins have been demonstrated to be more efficient pyrazine precursors than free amino acids.

Mechanistically, pyrazine formation is not governed by a single mechanistic pathway, but constitutes a complex network in which multiple pathways coexist and compete. The α-aminoketone condensation–oxidation pathway is the most prevalent route in sugar–amino acid systems; the radical pathway plays an important role in the early stages of the reaction; dehydration aromatisation provides an explanation for the mechanism of alkyl migration; the azomethine ylide dimerisation pathway offers a new route that does not depend on α-dicarbonyl compounds; and when peptides serve as precursors, they proceed *via* α-ketoimine intermediates rather than Strecker degradation. In recent years, the introduction of DFT computational chemistry has enabled researchers to advance from qualitative description of pathways to quantitative calculation of activation energy barriers and identification of rate-limiting steps.

The yield and distribution of pyrazines are synergistically regulated by multiple factors, including temperature, pH, precursor structure and reaction medium. Temperatures above 100 °C are generally required to effectively initiate the reaction, while alkaline conditions significantly promote pyrazine formation. With respect to precursor structure, the manner of sugar fragmentation determines the substitution pattern on the pyrazine ring, and peptide-type nitrogen sources exhibit much higher pyrazine-forming activity than free amino acids; however, excessively long peptide chains (pentapeptides and above) lose this activity. Organic co-solvents and emulsion systems can influence the reaction course by modulating substrate solubility and interfacial distribution. Emerging technologies, such as ultrasound, provide new technological routes for the efficient and targeted synthesis of pyrazines by lowering the activation energy barriers of the key steps in the Maillard reaction, whereas phenolic compounds and high-pressure treatment can inhibit pyrazine formation under specific conditions.

Based on the above understanding, future research directions may include the following:

(1) Structure–activity relationships between peptide structure and pyrazine products. Although peptides have been confirmed to be more efficient pyrazine precursors than free amino acids, how the amino acid sequence, spatial configuration of peptides, and the structure of peptide–sugar adduct intermediates specifically influence the distribution of substituents on the pyrazine ring remains to be systematically investigated. Elucidating this relationship would facilitate the prediction of pyrazine product profiles from peptide sequence information, providing a theoretical basis for the rational screening of peptide-based flavour precursors.

(2) Network analysis of multi-component interactions. In real food systems, sugars, amino acids, peptides, lipids and their degradation products coexist, and the synergistic and competitive effects among these components are not yet fully understood. Multi-omics approaches enable simultaneous tracking of the dynamic changes of hundreds of metabolites, while CAMOLA isotope labelling allows precise tracing of the origin and fate of carbon/nitrogen atoms. The integration of these two approaches offers a feasible technical pathway for deciphering the interactive networks of multi-component systems.

(3) Data-driven targeted synthesis of pyrazines. With the advancement of computational chemistry and machine learning methods, it will become possible to establish predictive models correlating precursor structure, reaction conditions and pyrazine product profiles. This direction holds the potential to transform the development of pyrazine flavour compounds from a trial-and-error approach to on-demand design, significantly improving R&D efficiency.

(4) Scale-up engineering and industrial applications. Current research is primarily conducted at the laboratory scale, and the feasibility and economic viability of engineering strategies such as continuous flow reactors and ultrasound-assisted processing await further validation. Once the engineering bottlenecks are overcome, the biomimetic synthesis of pyrazines, together with complementary microbial biosynthetic and catalytic routes, is expected to replace conventional chemical synthesis and natural extraction, becoming a new paradigm for the green manufacturing of food flavour compounds.^67,68^

## Author contributions

Bingyang Wang and Yao Zhao contributed equally. Bingyang Wang: supervision, writing – review & editing; Yao Zhao: investigation, formal analysis literature curation, writing – original draft; Lianchen Zhao: literature curation; Wentao Zhu: investigation; Jie Ren: investigation, formal analysis.

## Conflicts of interest

There are no conflicts of interest declared.

## Data Availability

No primary research results, software or code have been included and no new data were generated or analysed as part of this review.

## References

[cit1] Dawes I. W., Edwards R. A. (1966). Chem. Ind..

[cit2] Mason M. E., Johnson B., Hamming M. (1966). J. Agric. Food Chem..

[cit3] Bondarovich H. A., Friedel P., Krampl V., Renner J. A., Shephard F. W., Gianturco M. A. (1967). J. Agric. Food Chem..

[cit4] Wang P. S., Kato H., Fujimaki M. (1969). Agric. Biol. Chem..

[cit5] Maga J. A. (1982). CRC Crit. Rev. Food Sci. Nutr..

[cit6] Maga J. A. (1992). Food Rev. Int..

[cit7] Yu H., Zhang R. Y., Yang F. W., Xie Y. F., Guo Y. H., Yao W. R., Zhou W. B. (2021). Trends Food Sci. Technol..

[cit8] Zhou T., Cui H. P., Li X. J., Huang C., Askar G., Hayat K., Zhang X. M., Ho C. T. (2026). Food Chem..

[cit9] Ren A., Zhang Y., Bian Y., Liu Y. J., Zhang Y. X., Ren C. J., Zhou Y., Zhang T., Feng X. S. (2024). Food Chem..

[cit10] Koehler P. E., Odell G. V. (1970). J. Agric. Food Chem..

[cit11] Van Praag M., Stein H. S., Tibbetts M. S. (1968). J. Agric. Food Chem..

[cit12] Shibamoto T., Bernhard R. A. (1977). J. Agric. Food Chem..

[cit13] Adams A., Polizzi V., Van Boekel M. (2008). et al.. J. Agric. Food Chem..

[cit14] Ferretti A., Flanagan V. P., Ruth J. M. (1970). J. Agric. Food Chem..

[cit15] Hwang H. I., Hartman T. G., Rosen R. T., Ho C. T. (1993). J. Agric. Food Chem..

[cit16] Hwang H. I., Hartman T. G., Rosen R. T., Ho C. T. (1994). J. Agric. Food Chem..

[cit17] Deng S. B., Cui H. P., Hayat K., Zhai Y., Zhang Q., Zhang X. M., Ho C. T. (2022). Food Chem..

[cit18] Baltes W., Bochmann G. (1987). J. Agric. Food Chem..

[cit19] Shu C. K. (1999). J. Agric. Food Chem..

[cit20] Huang T. C., Bruechert L. J., Hartman T. G. (1987). et al.. J. Agric. Food Chem..

[cit21] Yu H., Seow Y. X., Ong P. K. C., Zhou W. B. (2018). npj Sci. Food.

[cit22] Hu B. B., Yin W. T., Yu T., Han Z. Q., Liu Y. X., Liu H. M., Cai X. S., Zhai Z. Q., Zhang F. (2025). Food Chem..

[cit23] Adams A., Kimpe N. D. (2009). Food Chem..

[cit24] Yu A. N., Zhang A. D. (2010). Food Chem..

[cit25] Yu A., Tan Z., Shi B. (2012). Asia-Pac. J. Chem. Eng..

[cit26] Van Lancker F., Adams A., De Kimpe N. (2010). J. Agric. Food Chem..

[cit27] Van Lancker F., Adams A., De Kimpe N. (2012). J. Agric. Food Chem..

[cit28] Wang F., Shen H. L., Liu T., Yang X., Yang Y. L., Guo Y. R. (2021). Foods.

[cit29] Scalone G. L. L., Cucu T., De Kimpe N. (2015). et al.. J. Agric. Food Chem..

[cit30] Scalone G. L. L., Lamichhane P., Cucu T., De Kimpe N., De Meulenaer B. (2019). Food Chem..

[cit31] Wang F., Shen H. L., Yang X., Liu T., Yang Y. L., Zhou X. R., Zhao P. T., Guo Y. R. (2021). RSC Adv..

[cit32] Chiang J. H., Yeo M. T. Y., Ong D. S. M., Henry C. J. (2022). Food Chem..

[cit33] Nishimura K., Abe T. (2025). Food Chem..

[cit34] Zou T., Liu J., Song H. (2018). et al.. J. Food Sci..

[cit35] Zou T. T., Kang L., Yang C., Song H. L., Liu Y. (2019). LWT–Food Sci. Technol..

[cit36] Scalone G. L. L., Ioannidis A. G., Lamichhane P. (2020). et al.. Food Res. Int..

[cit37] Yang C., Wang R., Song H. L. (2012). Food Chem..

[cit38] Newell J. A., Mason M. E., Matlock R. S. (1967). J. Agric. Food Chem..

[cit39] Gao P. X., Wang S., Feng B. M., Liu C. Y., Wang Y., Dou S. H., Dong L. (2025). Food Chem.:X.

[cit40] Amrani-Hemaimi M., Cerny C., Fay L. B. (1995). J. Agric. Food Chem..

[cit41] Shibamoto T., Bernhard R. A. (1976). J. Agric. Food Chem..

[cit42] Haider Shipar M. A. (2006). Food Chem..

[cit43] Hayashi T., Namiki M. (1981). Agric. Biol. Chem..

[cit44] Milić B. Lj., Piletić M. V. (1984). Food Chem..

[cit45] Guerra P. V., Yaylayan V. A. (2010). J. Agric. Food Chem..

[cit46] Ma Y. J., Wang X. Y., Zhu B. W., Du M., Dong L., Dong X. P., Xu X. B. (2022). Food Chem..

[cit47] González-Rodríguez J., Jurkaš V., Puchľová E., Podewitz M., Parmeggiani F., Winkler M., Both P., Šiška P., Rudroff F. (2026). Green Chem..

[cit48] Luo Y., Li R., Zhu S. Y., Huang Q. R., Xu B. J., Ho C. T. (2025). Food Chem..

[cit49] Lin P. X., Zhang H. J., Chen J. C. (2022). Food Sci..

[cit50] Jalbout A. F., Shipar M. A. H. (2007). Food Chem..

[cit51] Zhan H., Zhang L., Xu M. S., Yi X. Y., Li Y. G., Zhao Z. Z., Diao Y. D. (2025). Food Chem..

[cit52] Hua W. M., Liu H., Li M. Q., Tao R. M., Du J. Z., Gai S. M., Liu D. Y. (2026). Meat Res..

[cit53] Chu X. R., Wang X. H., Chen Y. Z., Du P. F., He H. J., Abd El-Aty A. M. (2025). J. Food Compos. Anal..

[cit54] Wong J. M., Bernhard R. A. (1988). J. Agric. Food Chem..

[cit55] Hwang H. I., Hartman T. G., Ho C. T. (1995). J. Agric. Food Chem..

[cit56] Xiao Q., Huang Q. R., Ho C. T. (2023). J. Agric. Food Chem..

[cit57] Yu X., Zhao M., Hu J. (2012). et al.. Food Chem..

[cit58] Troise A. D., Berton-Carabin C. C., Fogliano V. (2016). Food Chem..

[cit59] Xia X., Zhou T., Yu J. Y., Cui H. P., Zhang F. X., Hayat K., Zhang X. M., Ho C. T. (2023). J. Agric. Food Chem..

[cit60] Yuan Y. X., Hayat K., Cai J. B., Xu D., Xia S. Q., Cui H. P., Yu J. Y. (2025). J. Sci. Food Agric..

[cit61] Yu H., Seow Y. X., Ong P. K. C., Zhou W. B. (2018). Food Chem..

[cit62] Totlani V. M., Peterson D. G. (2005). J. Agric. Food Chem..

[cit63] Mildner-Szkudlarz S., Siger A., Szwengiel A., Przygonski K., Wojtowicz E., Zawirska-Wojtasiak R. (2017). Food Chem..

[cit64] Wang Z. Y., Cui H. P., Ma M. Y., Hayat K., Zhang X. M., Ho C. T. (2022). J. Agric. Food Chem..

[cit65] Zhang Y. Q., Yao Y. S., Zhou T., Zhang F. X., Xia X., Yu J. Y., Song S. Q., Hayat K., Zhang X. M., Ho C. T. (2023). J. Agric. Food Chem..

[cit66] Hill V. M., Isaacs N. S., Ledward D. A., Ames J. M. (1999). J. Agric. Food Chem..

[cit67] Tong W., Wang Y., Yang Y., Zou Y., Luo H., Pu J. (2025). Curr. Res. Food Sci..

[cit68] Wang B., Zhao Y., Chen W., Zhao L., Yang Q., Ren J., Wang G., Wang X. (2026). RSC Adv..

